# Long-lived triplet charge-separated state in naphthalenediimide based donor–acceptor systems†

**DOI:** 10.1039/d1sc00285f

**Published:** 2021-02-26

**Authors:** Alexander Aster, Christopher Rumble, Anna-Bea Bornhof, Hsin-Hua Huang, Naomi Sakai, Tomáš Šolomek, Stefan Matile, Eric Vauthey

**Affiliations:** Department of Physical Chemistry, University of Geneva CH-1211 Geneva Switzerland eric.vauthey@unige.ch; Department of Organic Chemistry, University of Geneva CH-1211 Geneva Switzerland; Department of Chemistry, University of Basel St. Johanns-Ring 19 Basel 4056 Switzerland

## Abstract

1,4,5,8-Naphthalenediimides (NDIs) are widely used motifs to design multichromophoric architectures due to their ease of functionalisation, their high oxidative power and the stability of their radical anion. The NDI building block can be incorporated in supramolecular systems by either core or imide functionalization. We report on the charge-transfer dynamics of a series of electron donor–acceptor dyads consisting of a NDI chromophore with one or two donors linked at the axial, imide position. Photo-population of the core-centred π–π* state is followed by ultrafast electron transfer from the electron donor to the NDI. Due to a solvent dependent singlet–triplet equilibrium inherent to the NDI core, both singlet and triplet charge-separated states are populated. We demonstrate that long-lived charge separation in the triplet state can be achieved by controlling the mutual orientation of the donor–acceptor sub-units. By extending this study to a supramolecular NDI-based cage, we also show that the triplet charge-separation yield can be increased by tuning the environment.

## Abstract 2

Ultrafast electron transfer from singlet and triplet excited states in equilibrium results in the population of both singlet and triplet charge-separated states.

## Graphical abstract

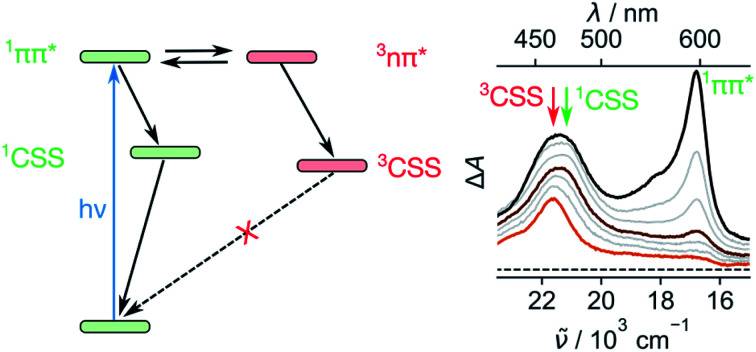

## 1 Introduction

Due to their high stability, good solubility and ease of functionalization, 1,4,5,8-naphthalenediimides (NDIs) are among the most used molecular building blocks in supramolecular chemistry.^1–7^ Their high oxidative power and the good stability of their radical anions make NDIs popular electron accepting units in multichromophoric systems.^8–26^ In principle, they can also act as chromophores due to their strong π–π* transition around 375 nm. In contrast to their bigger brother, the perylenediimide,^27^ unsubstituted NDIs have a low fluorescence quantum yield and a very short S_1_ state lifetime arising from a fast intersystem crossing (ISC).^28–31^ In agreement with the El Sayed's rule,^32^ the ISC is facilitated by a low lying T_*n*_ state of *n*–π* character, which enables a change of the spin angular momentum thanks to a concomitant change of orbital angular momentum.^28,33^ We have recently shown that, directly after photexcitation to the S_1_(π–π*) state, an equilibrium between this state and the almost isoenergetic T_*n*_(*n*–π*) state is established before the population is funnelled down to the T_1_(π–π*) state *via* internal conversion (IC).^34^

The photophysics of the electron deficient NDI can change drastically upon chemical functionalization with electron donors (Ds), depending on the site of modification. Core substitution makes the whole colour palette accessible due to the presence of a charge transfer (CT) state below the NDI-centred π–π* state.^35–37^ Since this CT state is located below the T_*n*_(*n*–π*) state, ISC is no longer allowed and the fluorescence lifetime increases from the ps to the ns regime.^35–37^

On the other hand, imide functionalization of NDIs with Ds has only a negligible impact on the absorption spectrum and reduction potential due to a weaker coupling to the NDI core.^2,4,28,33^ The high transition energy in the near UV, paired with the high oxidative power already in the ground state, renders the S_1_(π–π*) state of NDI a very strong electron acceptor (A) for photoinduced electron transfer (PET) reactions. As a consequence, PET is energetically feasible with relatively weak electron donors such as benzene, which is often used to axially link the NDI core to other chromophores or catalysts in multichromophoric systems.^21,38–40^

Even though imide functionalized NDIs are extensively used in model systems for artificial photosynthesis,^8,10,11,14,18–21^ and other supramolecular chemistry,^41–46^ their complex excited-state dynamics and the mechanistic cascade following PET are not fully understood.^33^ The main reason for this is that, in most cases, the NDIs are intended to serve as electron accepting units only. However, as they can absorb the high-energy side of the solar spectrum, they can also act as chromophores and, thus, their PET dynamics need to be better understood.

**Fig. 1:**
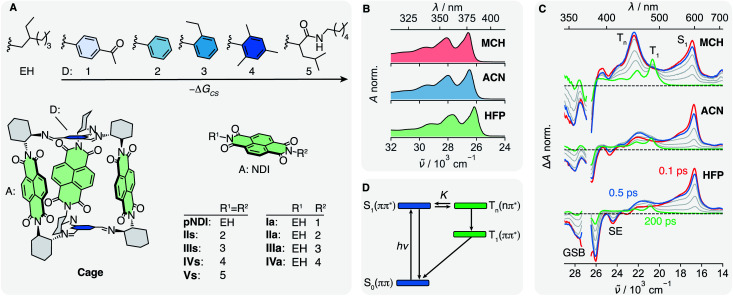
(A) Electron donors (D) 1–5 and ethyl hexyl (EH) are linked to the naphthalenediimide (NDI) electron acceptor (A) giving the symmetric dyads **IIs–Vs**, the asymmetric dyads **Ia–IVa** and the reference compound **pNDI**. In **Cage**, three NDIs are linked *via* an aromatic imine bridge, which acts as D, to a covalent organic cage. Absorption spectra (B) and transient absorption spectra (C) of **pNDI** in methylcyclohexane (MCH), acetonitrile (ACN) and hexafluoropropanol (HFP), upon excitation at 375 nm. (D) Energy-level scheme summarising the dynamics upon photo-population of the S_1_ state of **pNDI**. The relative energies of S_1_ and T_*n*_ hence the equilibrium constant *K*, depend on the solvent.

Herein we report on our investigation of axially-linked NDIs, aiming at a comprehensive picture of their excited-state dynamics. For this, we prepared a series of donor–acceptor (DA) dyads in which a NDI core is symmetrically (s) or asymmetrically (a) linked to 5 different Ds (Fig. 1A). The oxidation potential of the phenyl-based Ds decreases from 1 to 4, allowing –Δ*G*_CS_, the driving force for charge separation (CS), to be varied from <0.35 to about 0.85 eV,^47^ while keeping the coupling to the NDI core nearly constant. This series is compared with the reference NDI (**pNDI**), which is decorated with two innocent ethyl-hexyl (EH) linkers as well as to **Vs** with two non-aromatic and weakly coupled Ds 5. By applying transient-absorption spectroscopy in the UV-Vis and mid-infrared (IR), we will show that the ISC to the triplet manifold depends on the solvent and allows for the population of a long-lived triplet charge-separated state (^3^CSS) from the triplet T_*n*_(*n*–π*) state. Based on these results, we reinterpret the excited-state dynamics of a recently studied multichromophoric **Cage**,^45^ and show how the yield of the long-lived ^3^CSS can be enhanced by supramolecular chemistry.

## 2 Results and discussion

### 2.1 Solvent-dependent intersystem crossing of **pNDI**

We address the solvent dependence of the ISC dynamics of **pNDI**, which first needs to be clarified for understanding the more complex dyads. As expected for the π–π* transition of a symmetric chromophore, the stationary electronic absorption spectra of **pNDI** are nearly identical in solvents of different polarity and only a small red shift is observed in the strong hydrogen-bonding hexafluoro-2-propanol (HFP) (Fig. 1B and S1A†), pointing to stronger H-bond interactions in the excited than in the ground state.^48^ However, the transient absorption (TA) spectra, which report on excited-state processes occurring after excitation to the S_1_(π–π*) state at 375 nm, differ significantly (Fig. 1C). In acetonitrile (ACN), the excited-state absorption (ESA) band around 600 nm, the ground-state bleach (GSB), and the stimulated emission (SE) observed in the early spectra are indicative of the photo-population of the bright S_1_(π–π*) state. The latter decays partially within the first few hundreds of fs, whereas a band around 450 nm rises, which can be attributed to a T_*n*_(*n*–π*) state.^34^ Both S_1_ and T_*n*_ spectral features then decay simultaneously, and the T_1_(π–π*) band appears with its characteristic vibronic progression.^49^ These dynamics can be explained by a S_1_-T_*n*_ equilibrium established faster than the IC from the T_*n*_ to the T_1_ state (Fig. 1D).^34^ The T_*n*_ to S_1_ band-intensity ratio at equilibrium, *i.e.* after about 2 ps, decreases from 1.4 to 0.7 and 0.4 upon going from methylcyclohexane (MCH) to ACN and HFP, respectively (Fig. S9–S11†). Given that these ultrafast transitions occur on a similar timescale as those of vibrational/solvent relaxation, a true equilibrium constant cannot be defined.^50^ Therefore, *K* is only used here on a qualitative basis.

A similar dependence on the solvent polarity and hydrogen-bonding affinity was found with naphthalenemonoimides and attributed to the different electronic character of the two states involved in the equilibrium.^28,51^ Solvent polarity as well as hydrogen-bonding interactions destabilise the T_*n*_(*n*–π*) state relative to the S_1_(π–π*) state, allowing for a control of the equilibrium constant, *K*.^52–54^ Therefore, the equilibrium is shifted toward the S_1_(π–π*) state when going from MCH to ACN and HFP and *K* decreases accordingly. This trend is corroborated by measurements in the intermediate polarity solvents dibutylether (NBE) and tetrahydrofuran (THF) (Fig. S1B†). The solvent dependence of *K* additionally manifests as a slowing down of the concurrent decay of the S_1_ and T_*n*_ bands from MCH to ACN and HFP (Fig. S9–S11†). This can be explained using the pre-equilibrium approximation, 1/*τ*_obs_ ∝ *Kk*^T^_IC_, where *τ*_obs_ is the decay time of the S_1_ and T_*n*_ bands after equilibration and *k*^T^_IC_ is the rate constant of the T_*n*_ → T_1_ IC.

Even though the instantaneous population of the T_*n*_(*n*–π*) state is relatively low in polar and protic solvents, the faster T_*n*_ → T_1_ IC compared to the S_1_ → S_0_ IC funnels a substantial fraction of the excited-state population toward the T_1_ state (Fig. 1D). Triplet quantum yields for the population of the T_1_ state of 0.95, 0.7 and 0.5 in MCH, ACN and HFP, respectively, were estimated from the partial decrease of the GSB (ESI Section S4†). However, these values should be considered with some caution as the T_1_ state also absorbs in the GSB region (Fig. S2–S4†). Despite this, these triplet yields smaller than 1, together with the presence of a hot-ground state feature on the red side of the GSB in the TA spectra (Fig. S9–S10†), suggest the existence of an ultrafast deactivation channel to the ground state competing with ISC. Such an ultrafast non-radiative decay of a π–π* state was recently reported for a disulfide core-substituted NDI.^55^

In addition to the effect of solvent polarity on *K*, it is also important to note that the high oxidative power of the excited **pNDI**, *E*_red_(**pNDI**_S1_) = −3.7 V *vs.* SCE,^36^ enables PET from usually inert solvents such as toluene, dimethylsulfoxide or dimethylformamide. If these solvents are used in organic synthesis, excitation to the S_1_(π–π*) state should therefore be prevented to avoid the formation of radicals, possibly leading to unwanted side products.

### 2.2 Charge separation in **I–IV**

The π–π* absorption band of the NDI core remains almost unchanged upon covalent addition of Ds at the imide position. However, the bandwidth increases significantly with –Δ*G*_CS_ and the number of Ds (Fig. S5†). This band broadening was recently shown to originate from the ultrafast decay of the NDI-centred π–π* excited state upon CS.^47^

**Fig. 2:**
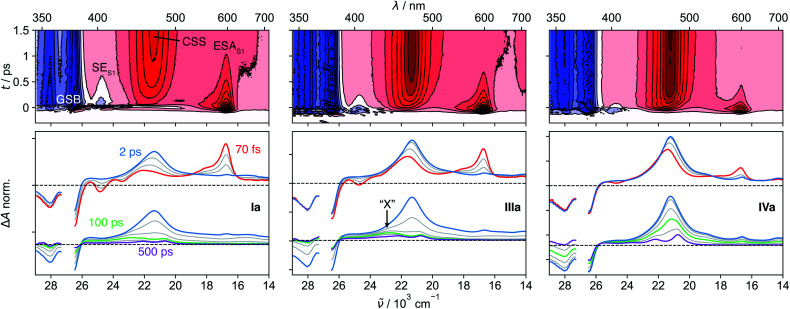
Transient absorption spectra measured with **Ia**, **IIIa** and **IVa** in acetonitrile. The contour plots in the top row illustrate the sub-ps decay of the excited-state absorption (ESA) and stimulated emission (SE) of the S_1_ state together with the rise of the charge-separated state (CSS) band. The spectral changes upon charge separation and charge recombination are illustrated in the bottom panel. The band “X” is visible with **Ia** and **IIIa** at around 100 ps.

The spectral features observed with **pNDI**, namely the ESA and the SE bands of the S_1_ state, are also present in the TA spectra measured directly after excitation of the dyads at 375 nm (Fig. 2). These bands, however, decay on the sub-ps timescale together with the rise of the NDI radical anion band at 470 nm,^56^ indicative of the population of the ^1^CSS upon charge separation. Although the ^1^CSS is the lowest singlet excited state of the dyads, S_1_ will be still used further on to designate the NDI-centred singlet π–π* state. The ultrafast CS dynamics of these dyads, measured by UV-Vis TA in ACN, were already discussed earlier.^47^ In brief, CS takes place with an average time constant ranging from 30 to 200 fs, depending on Δ*G*_CS_ and the number of Ds. CS accelerates with increasing driving force and is approximately twice as fast in the symmetric than the asymmetric dyads. Finally, the CS dynamics in MCH, measured with **IVs** only, was found to be the same as in ACN. For dyads **I–III**, an additional TA band “X” appears upon CS. This will be discussed in detail in Section 2.4.

**Fig. 3:**
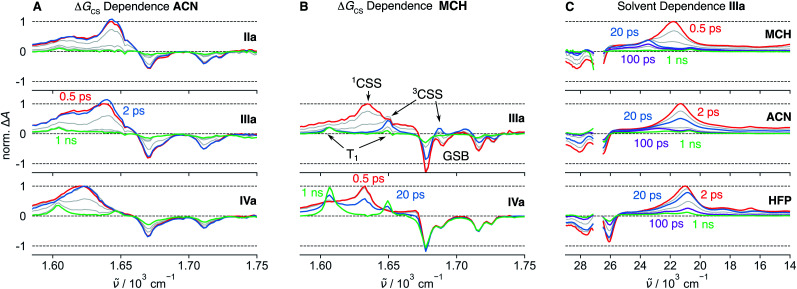
Mid-infrared transient absorption spectra in the symmetric and anti-symmetric imide carbonyl stretching region measured after excitation at 375 nm of dyads with different driving force for charge separation, −Δ*G*_CS_, in acetonitrile (ACN) (A) and methylcyclohexane (MCH) (B). (C) UV-Visible transient absorption spectra measured with **IIIa** in different solvents. The presence of the band “X” around 23 000 cm^−1^ follows the same solvent dependence as the S_1_(π–π*)–T_*n*_(*n*–π*) equilibrium constant *K* and is therefore attributed to the triplet charge-separated state.

TA measurements in the mid-infrared (IR) region reveal that the charge distribution in the ^1^CSS also depends on Δ*G*_CS_. We monitored the symmetric and antisymmetric stretches of the imide carbonyls, which are sensitive to subtle changes of the charge distribution^57–59^ that are otherwise not visible in the broad transient electronic absorption bands. As illustrated in Fig. 3A, the splitting of the two IR bands of the ^1^CSS located between 1600 and 1650 cm^−1^ decreases with increasing driving force and vanishes for **IVa**. This effect is mainly due to the frequency downshift of the most intense band that can be attributed to an increased electronic density on the carbonyl groups. Consequently, these results suggest an increase of the extent of charge separation in the ^1^CSS with the driving force.^60^

### 2.3 Charge recombination to the T_1_ state in **IV**

The UV-Vis TA spectra measured with **IVa** and **IVs** show that the decay of the ^1^CSS band is accompanied by the appearance of the T_1_ band, pointing to triplet charge recombination (CR) of the ^1^CSS, *i.e.* charge recombination to the triplet manifold (Fig. 2). This usually spin-forbidden process is enabled here due to the spin–orbit charge-transfer intersystem crossing (SOCT-ISC) mechanism. If the frontier molecular orbitals, localised mainly on the D and A subunits, have nearly perpendicular orientation, the change of orbital angular momentum upon CR allows for a change of spin angular momentum.^61–65^ The branching ratio of the singlet CR, *i.e.* recombination to S_0_, and triplet CR can be estimated from the IR-TA spectra, where the ESA bands do not overlap with the GSB (Fig. 3A and B). In the apolar MCH, the GSB remains constant during the decay of the ^1^CSS, indicating quantitative triplet CR *via* SOCT-ISC, which, combined with the sub-ps CS, gives a quantitative triplet yield. On the other hand, the GSB in ACN decreases to about 50% of its initial value during the decay of the ^1^CSS, pointing to equally probable triplet and singlet CR. In addition, CR is four times as fast in MCH (∼30 ps) compared to ACN (∼120 ps), suggesting that the stabilisation of the ^1^CSS by polar solvation has a significant impact on the SOCT-ISC dynamics (Fig. S22–S27†). This agrees with CR in the Marcus normal region,^66^ which is expected to slow down with decreasing ^1^CSS–T_1_ gap.

Contrary to CS, the CR dynamics are identical for the symmetric and asymmetric dyads in both ACN and MCH (Fig. S6†). This indicates that CS leads to symmetry breaking even in low-polarity solvents, rendering the probability for CR identical in the symmetric and asymmetric species.

### 2.4 Origin of the band “X” in **I–III**

In contrast to the UV-Vis TA spectra measured with **IVa** and **IVs**, where only the S_1_, ^1^CSS and T_1_ bands are visible, those measured with the dyads **I–III** exhibit an additional band, “X”, around 430 nm that is clearly visible after about 100 ps (Fig. 2). This feature was already observed in a previous study, but its origin could not be established.^33^ The knowledge acquired above with the solvent dependence of the S_1_–T_*n*_ equilibrium of **pNDI** can be used to interpret this band “X”. It is the most intense in MCH, is weaker in ACN, and is not visible in HFP (Fig. 3C). The S_1_(π–π*)–T_*n*_(*n*–π*) equilibrium constant *K* shows a similar solvent dependence, *i.e.*, decreases from MCH to ACN and HFP. Until now, we have only considered the population of ^1^CSS from the S_1_(π–π*) state. However, given the fast S_1_–T_*n*_ equilibrium, the population of the triplet charge-separated state (^3^CSS) from the T_*n*_(*n*–π*) state, which is thermodynamically favourable, should also be considered. Based on the similar solvent dependence of band “X” and equilibrium constant *K*, we propose that this band originates from the ^3^CSS, itself populated upon CS from the T_*n*_ state, in competition with IC to the T_1_ state.

According to this hypothesis, the ^3^CSS yield should depend on *K*, the dynamics of equilibration and the CS rate constants from the S_1_ and T_*n*_ states. In MCH, the equilibrium is shifted towards the T_*n*_ state, leading to a significant population of the ^3^CSS. By contrast, the equilibrium in HFP is strongly shifted towards the S_1_ state, whose population undergo CS to the ^1^CSS.

The population of the T_*n*_ state prior to CS can be clearly distinguished by comparing the UV-Vis TA spectra of the dyads with those of **pNDI** (Fig. S8†). For **I**, **II** and **III**, a shoulder that resembles the T_*n*_ band observed with **pNDI** is apparent at early times on the high-energy side of the anion band. This feature is absent with **IV**, the only dyad for which no ^3^CSS band is observed even in the apolar MCH. The absence of ^3^CSS with **IV** can be accounted for by the faster CS from the S_1_ state, which outcompetes the equilibration and a significant population of the T_*n*_ state.

Due to a smaller signal-to-noise ratio in the mid-IR region, spectral features that can be attributed to the ^3^CSS are only visible in MCH, as illustrated by the spectrum at 20 ps in Fig. 3B. The carbonyl stretching bands of the ^3^CSS are shifted to higher frequencies compared to T_1_, with one band overlapping with the 1690 cm^−1^ GSB and the other with the T_1_ band at 1650 cm^−1^. The ^1^CSS of **I–III** can either recombine to the S_0_ or the T_1_ state *via* the SOCT-ISC mechanism, as already discussed for **IV**. The recombination of the ^3^CSS to the S_0_ takes place *via* the same mechanism. It is visible in the mid-IR (Fig. 3B), where the ^3^CSS features and the GSB decay in parallel, while the amplitude of the most intense T_1_ band remains constant. Rather counter-intuitively, the opening of the triplet CS channel to the ^3^CSS thereby results in a smaller T_1_ yield. This is evident by comparing how the GSB decreases for **IIIa** but not for **IVa** (Fig. 3B).

### 2.5 Aromatic *vs.* non-aromatic electron donor

**Fig. 4:**
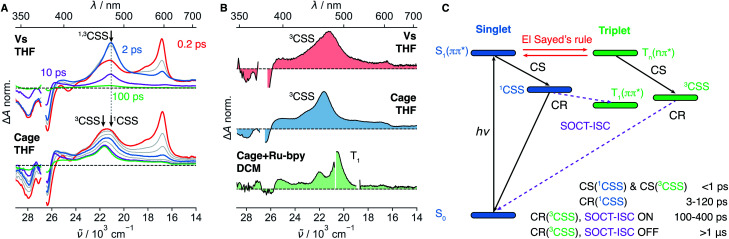
(A) Transient absorption spectra measured with **Vs** and **Cage** in tetrahydrofuran (THF) upon excitation at 375 nm. (B) Comparison of the residual signals (≥100 ps) of **Vs** and **Cage** attributed to the ^3^CSS and to the T_1_ state populated upon triplet sensitization of **Cage** with tris(bipyridine)ruthenium(ii) chloride (**Ru-bpy**). (C) Energy-level scheme summarising the dynamics upon photo-population of the S_1_(π–π*) state and timescales deduced from the transient absorption measurements (bottom). The spin–orbit charge-transfer intersystem crossing (SOCT-ISC) channel is only operative if the frontier molecular orbitals localised on the donor and acceptor sub-units are restricted to a near-perpendicular orientation.

To strengthen our assignment of band “X” to the ^3^CSS, we turned to dyad **Vs** in THF, where the non-aromatic D does not ensure a perpendicular arrangement of the frontier molecular orbitals. Similarly to the dyads with phenyl donors, the ^1^CSS band appears on the sub-ps timescale upon excitation of **Vs** to the S_1_ state (Fig. 4A and S28†). The ^1^CSS band does however decay significantly faster (∼5 ps) than with the other dyads and no T_1_ band is visible. As expected, SOCT-ISC is not operative and the ^1^CSS can only undergo singlet CR to the S_0_ state. Since CR occurs on the sub-10 ps timescale, hot ground-state features can be observed on the low-energy side of the GSB.^67^ As ground-state recovery upon CR is faster than vibrational relaxation, the instantaneous population of the vibrationally hot S_0_ state is large enough to be visible in the transient absorption spectrum. The faster CR compared to **I–IV** can be attributed to the non-aromatic character of the donor.^68,69^

Fig. 4A, B, S28 and S29† reveal that, after the sub-10 ps decay of the ^1^CSS band, a small residual signal, which does not decay on the ns timescale and is spectrally similar to the ^1^CSS, persists. The relative amplitude of this signal is slightly higher in the medium-polar THF than the highly-polar ACN and, thus, follows the same solvent dependence as the S_1_–T_*n*_ equilibrium constant *K* (Fig. S28–S29†). Triplet sensitization measurements reveal that this residual signal is spectrally different from the T_1_ absorption band of **Vs** (Fig. 4B). By analogy to the results obtained with **I–III**, the residual signal is attributed to the ^3^CSS. However, since the SOCT-ISC channel is not operative and CR of the ^3^CSS to the ground state is spin forbidden, the lifetime of the ^3^CSS is extended to a few μs at least (Fig. S31†). The slow decay of the ^3^CSS is biphasic, which could be due to the occurrence of a bimolecular process such as triplet–triplet annihilation or an interaction with some impurity. This decay was however not investigated in further detail, being outside the main scope of this study. The ^3^CSS yield could be expected to be larger in the non-polar MCH than in THF. Unfortunately, as **Vs** is not soluble in MCH, this could not be verified.

TA spectra recorded on the μs timescale do not exhibit the T_1_ band (Fig. S31†). This can be explained by a ^3^CSS located below the T_1_ state or by a small ^3^CSS–T_1_ gap, that results in a very slow CR to T_1_.

### 2.6 Increasing the ^3^CSS yield by supramolecular chemistry

Now, we want to illustrate with an example the importance of the above results for achieving long-lived charge separation. PET in a covalent organic cage (**Cage**, Fig. 1A) consisting of three NDI cores linked *via* an aromatic imine bridge was described recently.^45^ Upon excitation of a NDI sub-unit, an electron is transferred from the aromatic imine bridge to the NDI core. After CR, a residual spectral feature persisting on the ns timescale was attributed to the T_1_ state. This interpretation was based on the spectral and kinetic similarity with the T_1_ state of **pNDI** (Fig. 4B). Without the above results, we would have reached at the same conclusion.

We remeasured this cage upon 375 nm excitation in THF and could reproduce the TA spectra reported in ref. 45 (Fig. 4B). We furthermore carried out triplet sensitization measurements, as for **Vs**, which reveal that the long-lived spectral feature and the T_1_ spectrum of the cage are clearly different (Fig. 4B and S7†). Based on our results obtained with the dyads, we can re-interpret this long-lived spectral feature and assign it to the ^3^CSS. At early times, the CSS band is broader than that measured with **Vs** and consists of two partially overlapping bands. The low-frequency band matches that measured with **Vs**, decays within the first 10 ps and can thus be attributed to the ^1^CSS. The high-frequency band persists and is consequently assigned to the ^3^CSS, which, as for **Vs**, cannot recombine to the S_0_ state since this process is spin forbidden. The ^3^CSS yield is considerably higher for **Cage** than for **Vs** even though the same solvent was used. In view of the solvent dependence of the ^3^CSS yield found above, this suggests that the supramolecular architecture of **Cage** leads to a decrease of the local polarity and, thus, to a shift of the S_1_–T_*n*_ equilibrium towards the T_*n*_ state.^45^ Indeed, only three solvent molecules can occupy the restricted inner space of the **Cage**. As dipolar solvation is a long range interaction, the solvent field around the NDI cores is expected to strongly differ from that in bulk solvent. Additionally, the coupling between the three NDI sub-units can be expected to have a different effect on the two states in equilibrium. However, electrochemical measurements pointed to a weak interchromophoric coupling in **Cage**.^45^

Additional spectral features are visible on the μs timescale (Fig. S31†). They could arise from an intermolecular reaction of the ^3^CSS, and could be related to the sample degradation observed after prolonged laser irradiation. Although the decay of the ^3^CSS was not investigated in detail, these results indicate that it is slow enough to enable intermolecular reactions. This observation is highly relevant to photoredox catalysis with organic supramolecular cages that has emerged only very recently.^70,71^ The presence of the radical anion and the radical cation in the ^3^CSS state suggests that both reduction and oxidation reactions, respectively, could be achieved directly or photochemically, using a near IR light source around 800 nm that matches the lowest-energy absorption band of the NDI radical anion.^72,73^

### 2.7 Spectral differences between ^1^CSS and ^3^CSS

The positions of the ^1^CSS and ^3^CSS bands are nearly identical for **Vs**, slightly shifted for **Cage** and about 2000 cm^−1^ apart for the phenyl donor series. In principle, the electronic absorption spectra of ^1^CSS and ^3^CSS should be identical in the limit of zero electronic coupling between the D and A moieties. However, if the coupling is not negligible, the exchange interaction lifts the degeneracy of these two states and renders them spectrally distinct. An inert alkyl group separates the D and NDI sub-units in **Vs** and leads to a weak coupling. This is experimentally observable as a slow CS and a negligible spectral difference between ^1^CSS and ^3^CSS. By contrast, the coupling is larger for the phenyl Ds, which are directly linked to the imide nitrogen. This results in a stabilisation of the ^3^CSS relative to the ^1^CSS and in a blue shift of the ^3^CSS band. The exchange interaction, which is responsible for the ^1^CSS–^3^CSS energy splitting, also affects the electronic distribution that can be probed by vibrational spectroscopy (Fig. 3B).

## 3 Conclusion

A comprehensive picture of the photophysics of axially modified NDIs could be drawn by systematically varying the N-substituted donors as well as the environment. Two channels, which efficiently connect the singlet to the triplet manifolds, were revealed and lead to rich photophysics (Fig. 4C). The efficiency of the first channel is due to the conservation of the overall angular momentum upon S_1_(π–π*) to T_*n*_(*n*–π*) transition, in agreement with the El-Sayed's rule, and to the energetic proximity of these two states, which is intrinsic to the NDI core. We have herein shown that an equilibrium is reached on the sub-ps timescale and the associated constant *K* strongly depends on the polarity and hydrogen-donating ability of the solvent. The relative population of the triplet state and *K* are the highest in apolar solvents, decrease in polar solvent and are the lowest in strong hydrogen-bond donating solvents. Upon linking a D at the axial imide position, PET occurs from both equilibrated singlet and triplet states. The relative ^1^CSS and ^3^CSS populations depends on *K* and on the respective CS rate constants. However, these processes are too fast and the spectra of the different species involved overlap too much to allow determining a rate constant for each of these CS pathways. The second singlet-triplet channel becomes active after population of the two CSS. It depends on the spatial arrangement of the NDI core and the Ds. With phenyl-based donors, the frontier molecular orbitals on the NDI core and D are arranged at near 90°, enabling CR of the ^1^CSS and ^3^CSS to the T_1_ and S_0_ states, respectively. Without such near-perpendicular dihedral angle between the NDI core and D planes, the SOCT-ISC channel is shut down and the lifetime of the ^3^CSS is significantly increased. The yield of the long-lived ^3^CSS can be controlled by the polarity of the solvent and is the highest in non-polar solvents. In addition, we have shown that the ^3^CSS yield can be enhanced by incorporating the NDI unit in a multichromophoric structure. These remarkable excited-state properties of NDIs could be further exploited by integrating these chromophores in molecular architectures developed for applications based on long-lived photoinduced charge separation.

## Conflicts of interest

There are no conflicts to declare.

## Supplementary Material

SC-012-D1SC00285F-s001
