# Supplementary material for: Long-lived triplet charge-separated state in naphthalenediimide based donor–acceptor systems
Source: Chem Sci. 2021 Feb 26;12(13):4908–15. doi: 10.1039/d1sc00285f (PMC8179635; doi:10.1039/d1sc00285f)
Supplement: SC-012-D1SC00285F-s001 [file SC-012-D1SC00285F-s001.pdf]

Electronic Supplementary Material (ESI) for Chemical Science.
This journal is © The Royal Society of Chemistry 2021

Long-lived triplet charge-separated state in naphthalenediimide based donor-acceptor systems.

Alexander Aster, Christopher Rumble, Anna-Bea Bornhof,
Hsin-Hua Huang, Naomi Sakai, Tomáš Šolomek, Stefan Matile, and Eric Vauthey*

Electronic Supplementary Information

Contents

S1 Synthesis . . . S2
S2 Experimental Details, Spectroscopy . . . S2
S2.1 Chemicals . . . S2
S2.2 Absorption . . . S2
S2.3 UV-Vis Transient Absorption . . . S2
S2.3.1 General Remarks . . . S2
S2.3.2 Visible Probe . . . S2
S2.3.3 fs-ps Pump . . . S2
S2.3.4 ps-µs Pump . . . S2
S2.3.5 Data Treatment . . . S2
S2.4 Mid-Infrared (IR) Transient Absorption . . . S3
S3 Solvent dependence of the photophysics of pNDI . . . S4
S4 Estimation of the triplet quantum yield of pNDI . . . S5
S5 Steady-state electronic absorption spectra of I-V . . . S6
S6 Electron-transfer dynamics of I-V in ACN . . . S7
S7 Triplet sensitization experiments . . . S8
S8 Transient absorption spectra measured with pNDI and I-IV in ACN . . . S9
S9 Global lifetime analysis and evolution associated difference spectra . . . S10
S9.1 General . . . S10
S9.2 fs-ps Vis-TA of **pNDI** in MCH . . . S10
S9.3 fs-ps Vis-TA of **pNDI** in ACN . . . S11
S9.4 fs-ps Vis-TA of **pNDI** in HFP . . . S11
S9.5 fs-ps Vis-TA of **Ia** in ACN . . . S12
S9.6 fs-ps Vis-TA of **IIa** in ACN . . . S13
S9.7 fs-ps IR-TA of **IIa** in ACN . . . S13
S9.8 fs-ps Vis-TA of **IIs** in ACN . . . S14
S9.9 fs-ps Vis-TA of **IIIa** in ACN . . . S15
S9.10 fs-ps IR-TA of **IIIa** in ACN . . . S15
S9.11 fs-ps Vis-TA of **IIIa** in MCH . . . S16
S9.12 fs-ps IR-TA of **IIIa** in MCH . . . S16
S9.13 fs-ps Vis-TA of **IIIa** in HFP . . . S17
S9.14 fs-ps Vis-TA of **IIIs** in ACN . . . S18
S9.15 fs-ps Vis-TA of **IVa** in ACN . . . S19
S9.16 fs-ps IR-TA of **IVa** in ACN . . . S19
S9.17 fs-ps Vis-TA of **IVa** in MCH . . . S20
S9.18 fs-ps IR-TA of **IVa** in MCH . . . S20
S9.19 fs-ps Vis-TA of **IVs** in ACN . . . S21
S9.20 fs-ps Vis-TA of **IVs** in MCH . . . S22
S9.21 fs-ps Vis-TA of **Vs** in ACN . . . S23
S9.22 fs-ps Vis-TA of **Vs** in THF . . . S24
S9.23 fs-ps Vis-TA of **Cage** in THF . . . S25
S10 ns-µs Vis-TA of Cage and Vs in THF . . . S26

S1 Synthesis

pNDI,[1] **Ia**,[2] **IIa**,[1] **IIs**,[3] **IIIa**,[4] **IIIs**,[4] **IVa**,[4] **IVs**,[3] **Vs**[5] and **Cage**[6] are known compounds and their NMR spectra were in agreement with the literature.

S2 Experimental Details, Spectroscopy

S2.1 Chemicals

Acetonitrile (ACN, Roth, $\geq$ 99.9%), methylcyclohexane (MHX, Roth, $\geq$ 99%), hexafluoro-2-propanol, (HFP, Sigma-Aldrich, $\geq$99%), tetrahydrofuran (THF, Roth $\geq$ 99%) and n-buthylether (NBE, $\geq$ 99%, Acros) were used as received.

S2.2 Absorption

Absorption spectra were measured on a Cary 50 spectrometer.

S2.3 UV-Vis Transient Absorption

S2.3.1 General Remarks

The visible transient absorption (TA) data presented in this work were recorded with a fs-ps visible (fs-VIS) and a ps-µs visible (ps-VIS) TA setups. A detailed description of the general principle of the fs-ps as well as ps-µs TA applying referenced detection with two spectrographs is presented elsewhere.[7] The designs of the fs-VIS and the ps-VIS probe beam path are identical. The experimental details concerning these two setups and the data analysis are discussed below. The absorbance of the samples at the excitation wavelength was 0.05-0.4 on 1 mm. The samples were measured in 1 mm quartz cuvettes (Starna, model 1GS/Q/1) and bubbled with nitrogen during the measurements giving a wavelength dependent instrument response function (IRF) of about 80-350 fs (FWHM of the optical Kerr effect (OKE)). The absorption spectra of all samples, except **Cage**, showed no sign of degradation after the experiments. **Cage** showed only minor changes after recording the TA data and no differences in the time profiles of the four recorded scans could be observed. However, photo degradation could be observed after prolonged laser irradiation.

S2.3.2 Visible Probe

Probing in both setups was achieved using white-light pulses generated by focusing the 800 nm pulses of the Ti:Sapphire amplified system in a CaF_2 plate. The experimental layout was the same as that described earlier,[8] except that all lenses, after white light generation, were replaced by spherical mirrors to prevent chromatic aberration.

S2.3.3 fs-ps Pump

Excitation was performed using 375 nm pulses generated by doubling the 750 nm output a TOPAS-Prime in combination with a NirUVis frequency mixer (both from Light Conversion), which were themselves seeded by the output of a 1 kHz Ti:Sapphire amplified system (Spectra Physics, Solstice Ace). The transient absorption signal was checked prior to the measurement to scale linearly with the pump intensity. The polarization of the pump pulses was set to magic angle relative to the white-light pulses.

S2.3.4 ps-µs Pump

The ps-µs pumping was described in detail in ref. 9. Excitation was performed at 532 nm or 355 nm using a passively Q-switched, frequency doubled Nd:YAG laser (Teem Photonics, Powerchip NanoUV) producing pulses with a 500 Hz repetition rate, approximately 20 μJ energy, and 300 ps duration.

S2.3.5 Data Treatment

The pixel to wavelength conversion was achieved using a standard containing rare earth metals (holmium oxide) which shows narrow bands from the UV to the visible spectral region. All transient absorption spectra were corrected for background signals showing up before time zero (e. g. spontaneous emission).
The fs-ps spectra were corrected for the dispersion due to the optical chirp using the optical Kerr effect.[10]

S2.4 Mid-Infrared (IR) Transient Absorption

Time resolved UV-pump IR-probe measurements were performed using a previously described instrument [11] with excitation pulses produced by a second optical parametric amplifier (OPA). Briefly, fundamental pulses at 800 nm were generated using a chirped-pulse amplified laser system (Spectra Physics Solstice). One fraction of the fundamental was used to generate excitation pulses at 375 nm using an OPA (TOPAS-C, Light Conversion), and a second fraction was used to generate IR probe pulses using an OPA (TOPAS-C Light Conversion) and non-collinear difference frequency mixing (NDFG, Light Conversion). Dispersion of the probe light was accomplished using a Triax 190 spectrograph (Horiba, 150 lines/mm) and detected using a 2 x 64 MCT array (Infrared Systems Development). Two measurements were performed from 1585-1700 cm^{-1} and 1635-1760 cm^{-1}. They were merged by averaging the overlapping spectral region. Samples were flowed through a 300 µm cell constructed of CaF_2 windows, and the absorbance of the samples at 375 nm was kept below 0.2 in this cell for all measurements.

S3 Solvent dependence of the photophysics of pNDI

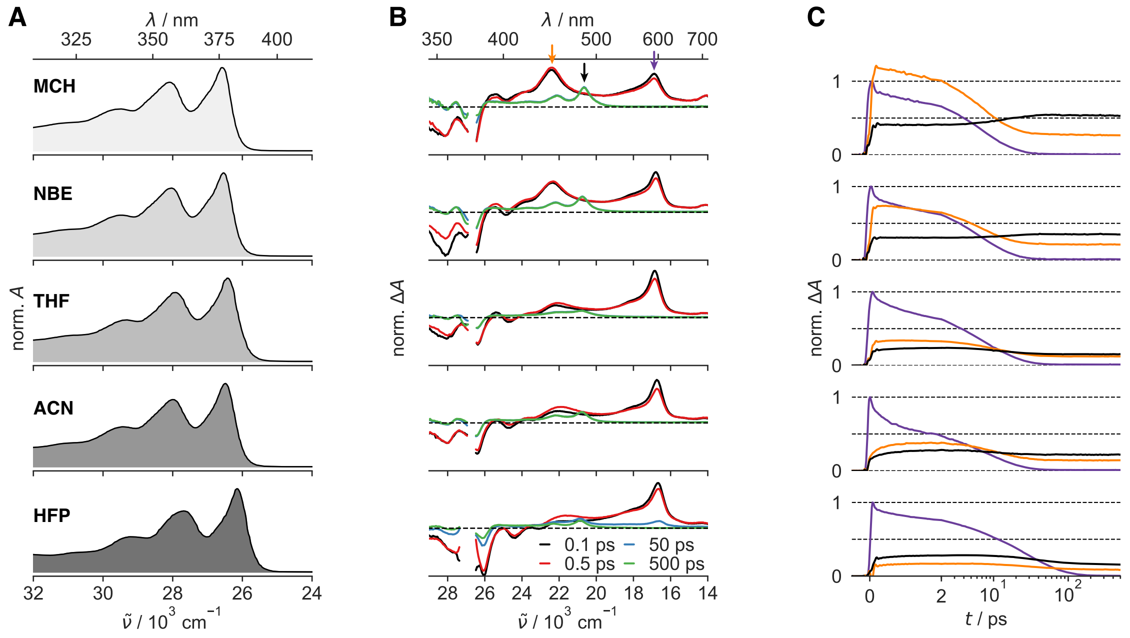

Figure S1: (A) Steady-state electronic absorption of **pNDI** in various solvents. (B) Transient absorption spectra at different time-steps showing the change in the S_1-T_n equilibrium. (C) Time profiles at the wavenumbers indicated in B.

S4 Estimation of the triplet quantum yield of pNDI

The triplet quantum yield of **pNDI** in different solvents, i.e. the yield for the T_1 state, was estimated from the visible transient absorption spectra at two time delays. First, the ground-state bleach (GSB) at 0.5 ps, where the population has not returned to the ground state (100 %), is filled up with the steady-state absorption spectrum until the spectral features of the GSB are no longer visible. Second, the same procedure is repeated at time delays, where the whole population resides in the T_1 state. Comparing the amplitudes of the steady-state absorption spectra required to fill up the GSB at the two time delays allows an estimation of the triplet yield. Neither the shape nor the intensity of the T_1 feature does change from the second time delay selected up to the 1.5 ns upper time limit of the experiment. This is due to the microsecond lifetime of the T_1 state. As illustrated in Figure S2-S4, the spectral feature of the T_1 state overlaps with the GSB. Note that the negative features at early times, which remain after filling up the GSB, arise from the stimulated emission.
The triplet yield of **pNDI** was estimated to be 50, 70 and 95% in HFP, ACN and MCH, respectively.

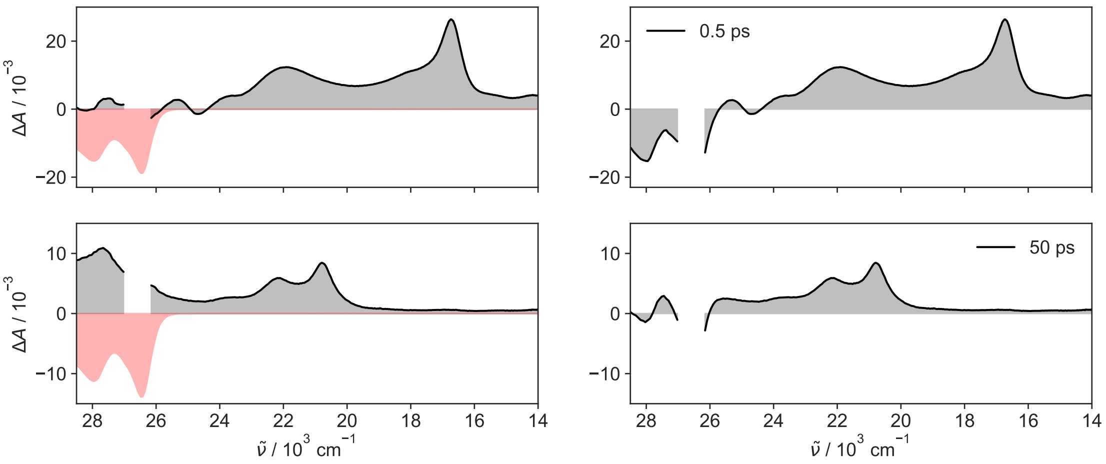

Figure S2: Determination of the triplet quantum yield of **pNDI** in ACN by filling up the ground-state bleach at early times (top panels), where nearly no excited-state population has yet decayed and at later times (lower panels) where the whole population resides in the T_1 state. The raw data is shown on the right, whereas the filled-up spectra (grey) with the steady-state absorption (red) are depicted on the left. A triplet yield of 70 % could be estimated.

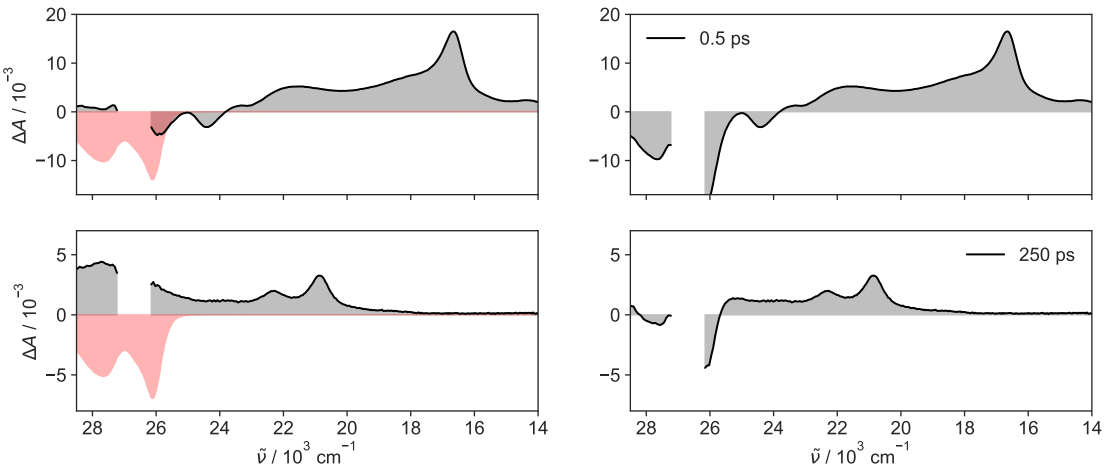

Figure S3: Determination of the triplet quantum yield of **pNDI** in HFP by filling up the ground state bleach at early times (top panels), where nearly no excited-state population has yet decayed and at later times (lower panels) where the whole population resides in the T_1 state. The raw data is shown on the right, whereas the filled-up spectra (grey) with the steady-state absorption (red) are depicted on the left. A triplet yield of 50 % could be estimated.

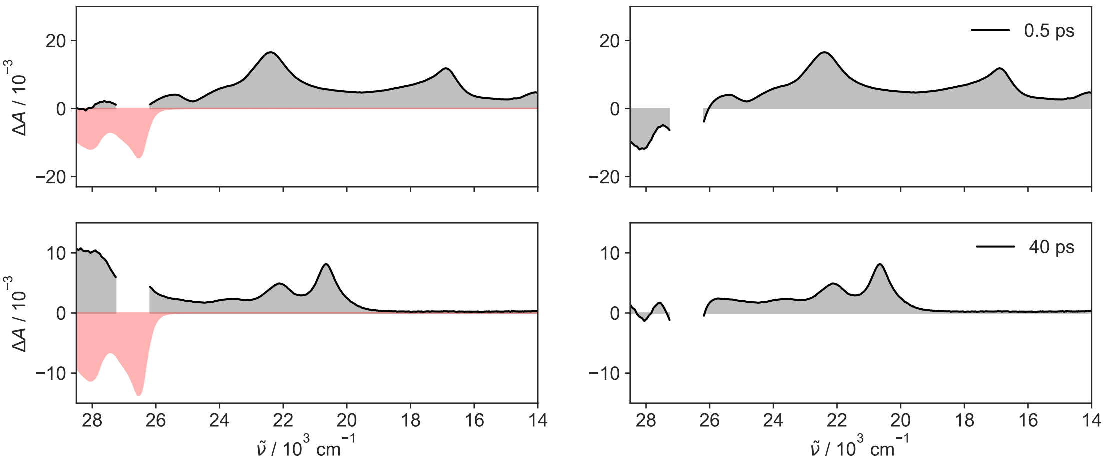

Figure S4: Determination of the triplet quantum yield of **pNDI** in MCH by filling up the ground state bleach at early times (top panels), where nearly no excited-state population has yet decayed and at later times (lower panels) where the whole population resides in the T_1 state. The raw data is shown on the right, whereas the filled-up spectra (grey) with the steady-state absorption (red) are depicted on the left. A triplet yield of 95 % could be estimated.

S5 Steady-state electronic absorption spectra of I-V

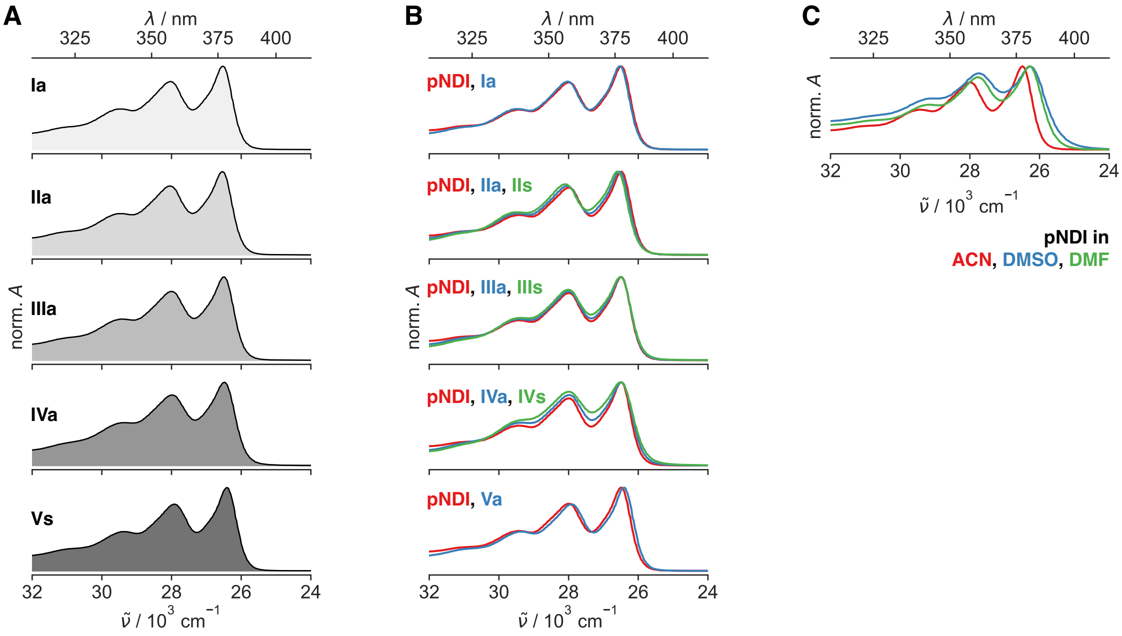

Figure S5: (A) Comparison of the steady-state electronic absorption spectra of the NDI-D dyads in acetonitrile (ACN). (B) Change in absorption spectrum with the number of electron donors. (C) Broadening of the absorption spectrum in DMSO and DMF in which photoinduced electron transfer to the solvent occurs.

S6 Electron-transfer dynamics of I-V in ACN

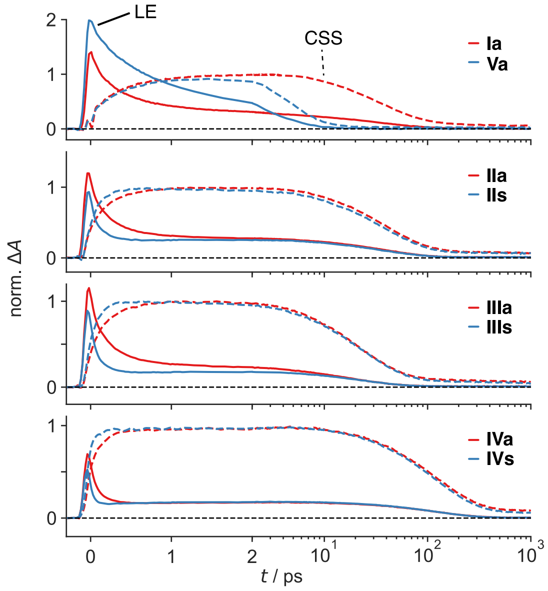

Figure S6: Time profiles measured with the different dyads in acetonitrile at the maximum of the S_1 ESA band (LE, solid line) and of the charge-separated state band (CSS, dashed line).

S7 Triplet sensitization experiments

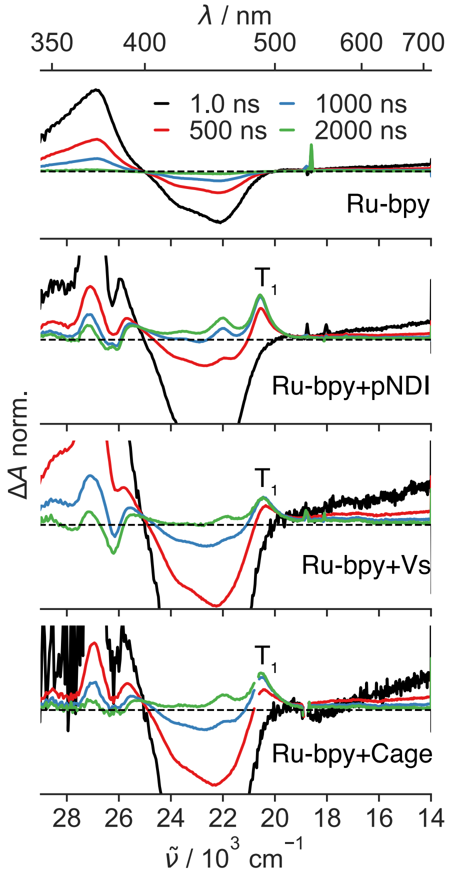

Figure S7: Ps-µs transient absorption spectra measured upon 532 nm excitation of ruthenium-tris(2,2'-bipyridyl) dichloride (Ru-bpy) in dichloromethane. If the presence of **pNDI**, **Vs** or **Cage** in the dichloromethane solution of Ru-bpy, the $T_1(\pi$-$\pi^*)$ state of NDI is populated via triplet energy transfer. It can be clearly seen that, in contrast to the ^{3}CSS band, the T_1 band shows a vibronic progression.

S8 Transient absorption spectra measured with pNDI and I-IV in ACN

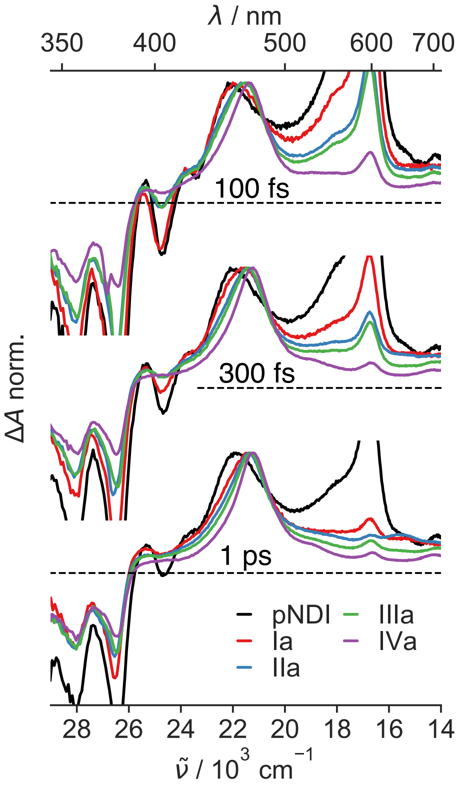

Figure S8: Comparison of transient absorption spectra in acetonitrile at different time delays. The spectra are normalized to the maximum in the spectral region between 20000 and 24000 cm^{-1}.

S9 Global lifetime analysis and evolution associated difference spectra

S9.1 General

The broadband transient absorption data were analysed using global lifetime analysis.[12] We assumed a sequential model for all the datasets analysed. This corresponds to a single population that evolves as a series of n successive exponential steps into further species without any losses or back-reactions. This analysis yields the so-called Evolution-Associated Difference Spectra (EADS), which represent the spectral evolution. However, the EADS do not systematically correspond to given species or states, but only help to visualize the timescales on which the spectral dynamics occur. Especially, the sub 100 ps lifetimes include contribution from both population transfer and relaxation processes.

The data after 0.5 ps was used for the analysis in the IR as well as UV-Vis spectral region. Therefore, charge separation is only partly captured and the extracted lifetimes are not used to describe population transfer to the charge-separated state. The charge separation dynamics were recently discussed in detail elsewhere.[4]

S9.2 fs-ps Vis-TA of pNDI in MCH

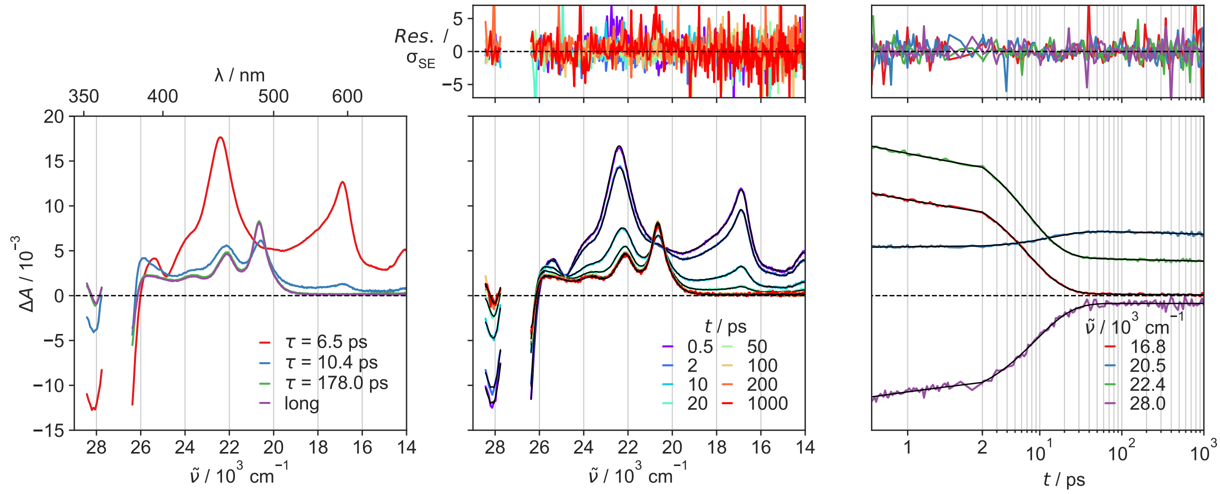

Figure S9: Global lifetime analysis of the UV-Vis transient absorption data measured with **pNDI** in methylcyclohexane on the fs-ps timescale upon excitation at 375 nm. Left panel, evolution-associated difference spectra (EADS) and respective time constants. Middle and right panels, comparison of the best-fit (black line) and the spectra and time profiles at given time delays and wavenumbers, respectively.

S9.3 fs-ps Vis-TA of pNDI in ACN

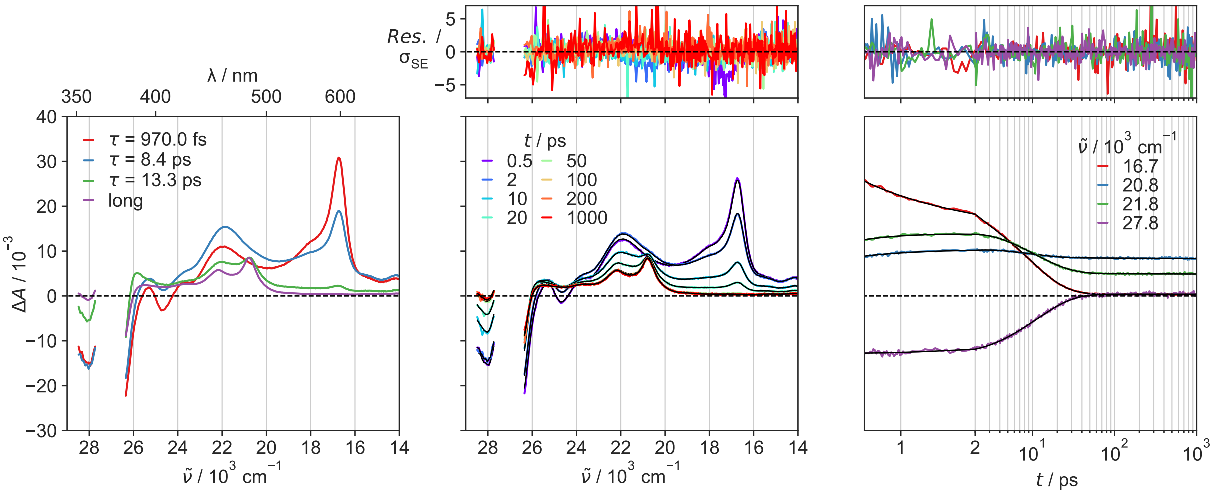

Figure S10: Global lifetime analysis of the UV-Vis transient absorption data measured with **pNDI** in acetonitrile on the fs-ps timescale upon excitation at 375 nm. Left panel, evolution-associated difference spectra (EADS) and respective time constants. Middle and right panels, comparison of the best-fit (black line) and the spectra and time profiles at given time delays and wavenumbers, respectively.

S9.4 fs-ps Vis-TA of pNDI in HFP

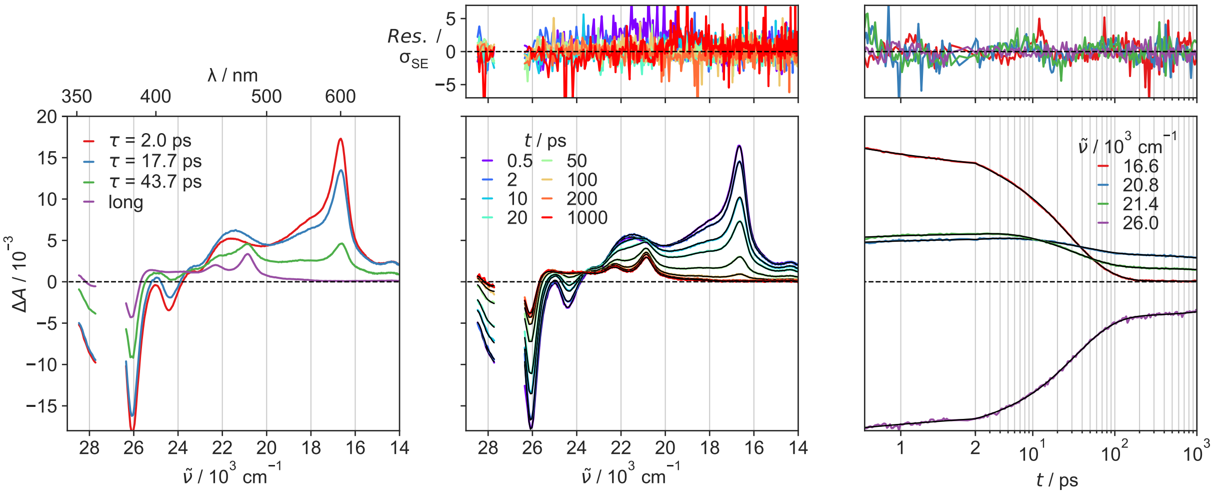

Figure S11: Global lifetime analysis of the UV-Vis transient absorption data measured with **pNDI** in hexafluoropropanol on the fs-ps timescale upon excitation at 375 nm. Left panel, evolution-associated difference spectra (EADS) and respective time constants. Middle and right panels, comparison of the best-fit (black line) and the spectra and time profiles at given time delays and wavenumbers, respectively.

S9.5 fs-ps Vis-TA of Ia in ACN

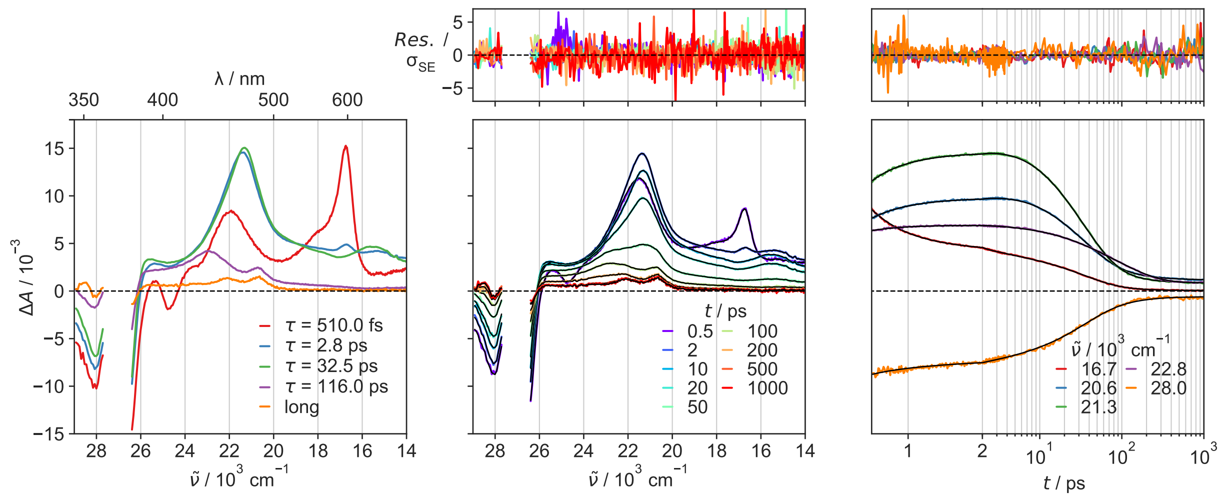

Figure S12: Global lifetime analysis of the UV-Vis transient absorption data measured with **Ia** in acetonitrile on the fs-ps timescale upon excitation at 375 nm. Left panel, evolution-associated difference spectra (EADS) and respective time constants. Middle and right panels, comparison of the best-fit (black line) and the spectra and time profiles at given time delays and wavenumbers, respectively.

S9.6 fs-ps Vis-TA of IIa in ACN

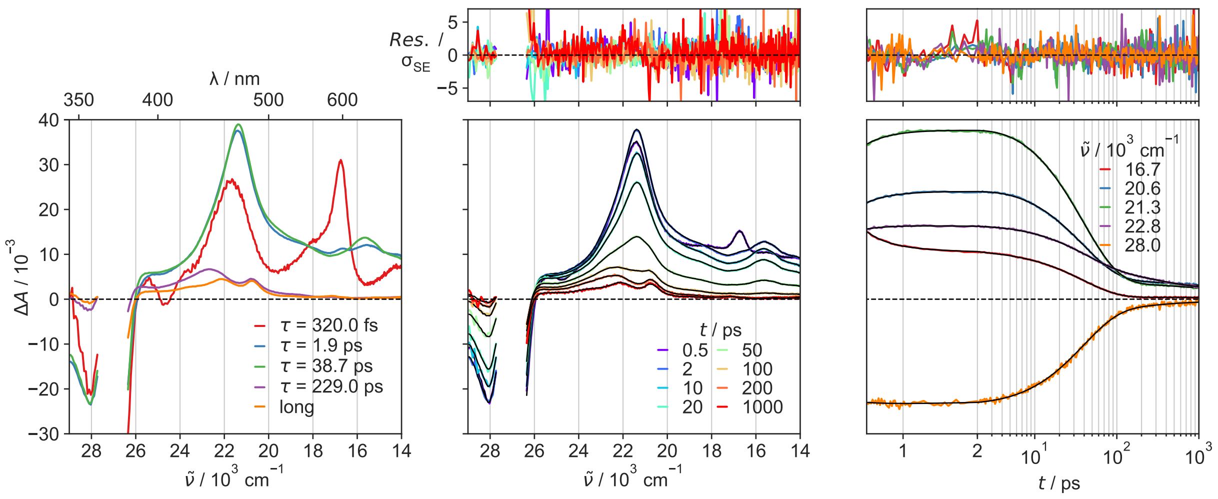

Figure S13: Global lifetime analysis of the UV-Vis transient absorption data measured with **IIa** in acetonitrile on the fs-ps timescale upon excitation at 375 nm. Left panel, evolution-associated difference spectra (EADS) and respective time constants. Middle and right panels, comparison of the best-fit (black line) and the spectra and time profiles at given time delays and wavenumbers, respectively.

S9.7 fs-ps IR-TA of IIa in ACN

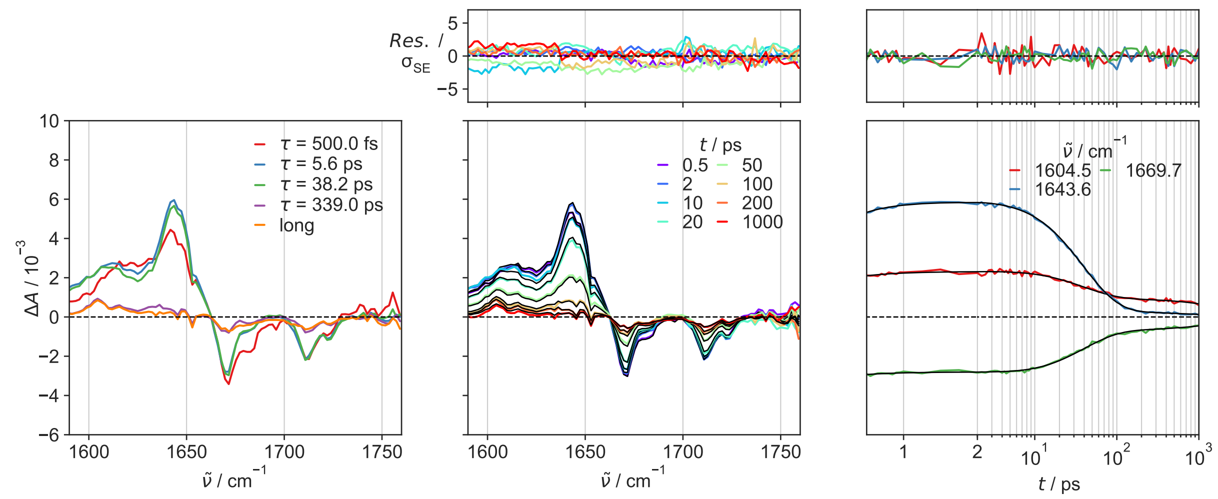

Figure S14: Global lifetime analysis of the mid-IR transient absorption data measured with **Ia** in acetonitrile on the fs-ps timescale upon excitation at 375 nm. Left panel, evolution-associated difference spectra (EADS) and respective time constants. Middle and right panels, comparison of the best-fit (black line) and the spectra and time profiles at given time delays and wavenumbers, respectively.

S9.8 fs-ps Vis-TA of IIs in ACN

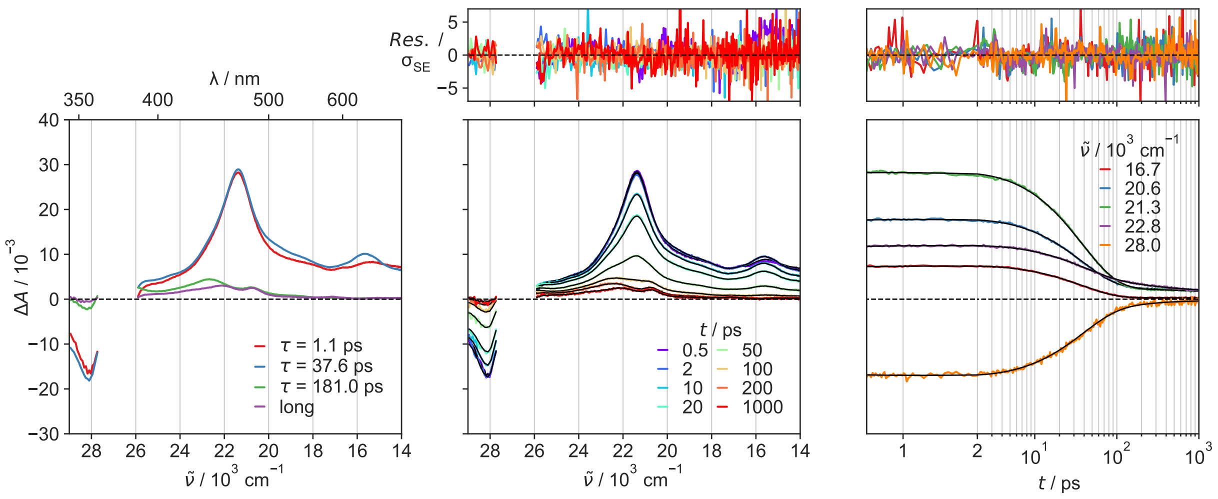

Figure S15: Global lifetime analysis of the UV-Vis transient absorption data measured with **IIs** in acetonitrile on the fs-ps timescale upon excitation at 375 nm. Left panel, evolution-associated difference spectra (EADS) and respective time constants. Middle and right panels, comparison of the best-fit (black line) and the spectra and time profiles at given time delays and wavenumbers, respectively.

S9.9 fs-ps Vis-TA of IIIa in ACN

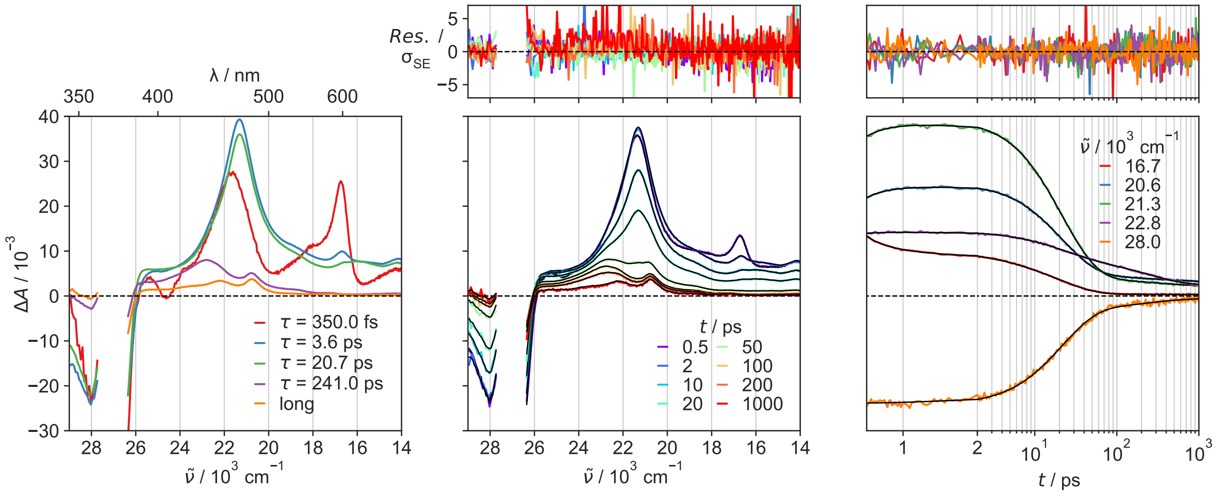

Figure S16: Global lifetime analysis of the UV-Vis transient absorption data measured with **IIIa** in acetonitrile on the fs-ps timescale upon excitation at 375 nm. Left panel, evolution-associated difference spectra (EADS) and respective time constants. Middle and right panels, comparison of the best-fit (black line) and the spectra and time profiles at given time delays and wavenumbers, respectively.

S9.10 fs-ps IR-TA of IIIa in ACN

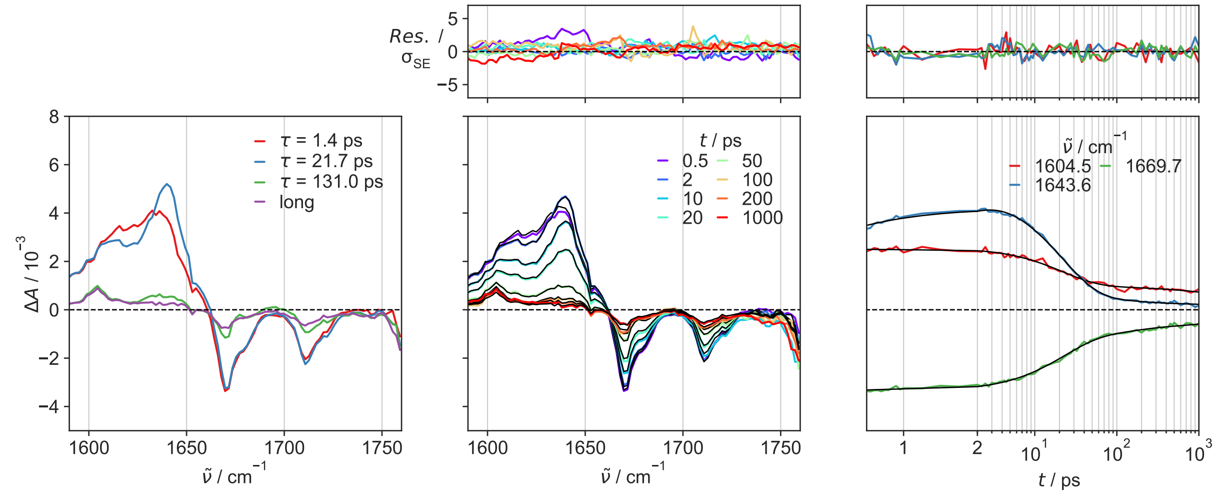

Figure S17: Global lifetime analysis of the mid-IR transient absorption data measured with **IIIa** in acetonitrile on the fs-ps timescale upon excitation at 375 nm. Left panel, evolution-associated difference spectra (EADS) and respective time constants. Middle and right panels, comparison of the best-fit (black line) and the spectra and time profiles at given time delays and wavenumbers, respectively.

S9.11 fs-ps Vis-TA of IIIa in MCH

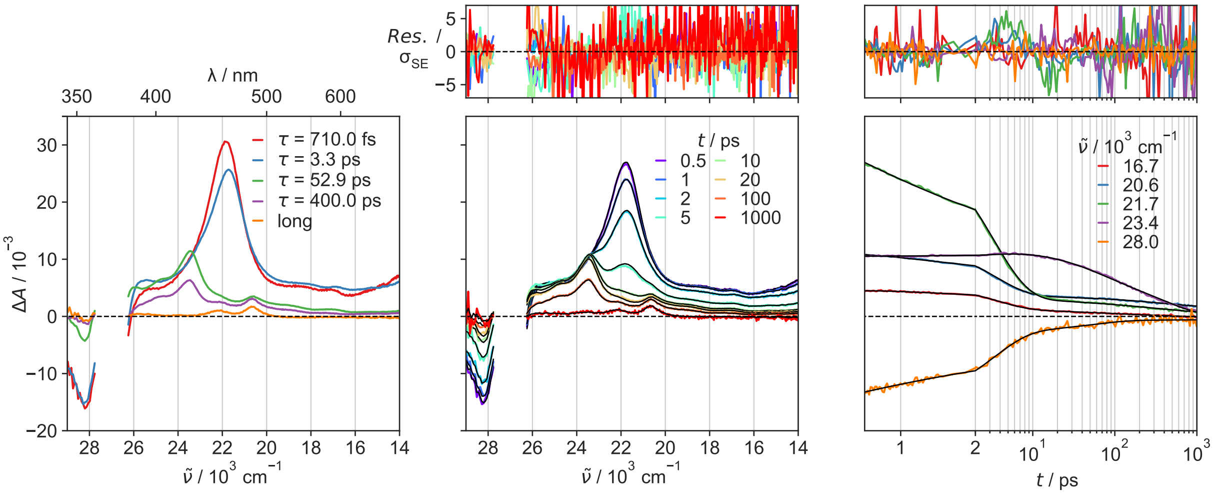

Figure S18: Global lifetime analysis of the UV-Vis transient absorption data measured with **IIIa** in methylcyclohexane on the fs-ps timescale upon excitation at 375 nm. Left panel, evolution-associated difference spectra (EADS) and respective time constants. Middle and right panels, comparison of the best-fit (black line) and the spectra and time profiles at given time delays and wavenumbers, respectively.

S9.12 fs-ps IR-TA of IIIa in MCH

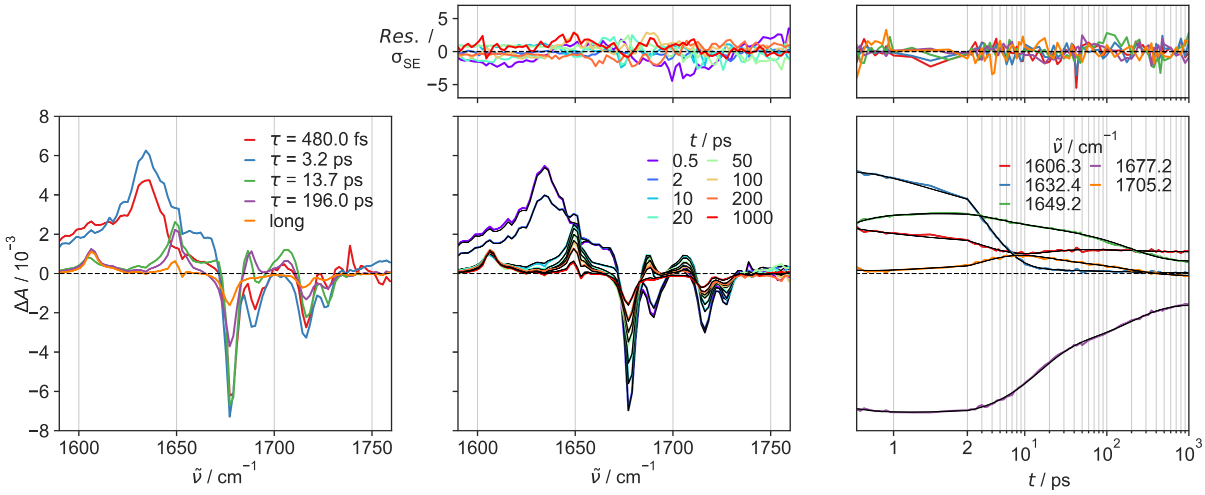

Figure S19: Global lifetime analysis of the mid-IR transient absorption data measured with **IIIa** in methylcyclohexane on the fs-ps timescale upon excitation at 375 nm. Left panel, evolution-associated difference spectra (EADS) and respective time constants. Middle and right panels, comparison of the best-fit (black line) and the spectra and time profiles at given time delays and wavenumbers, respectively.

S9.13 fs-ps Vis-TA of IIIa in HFP

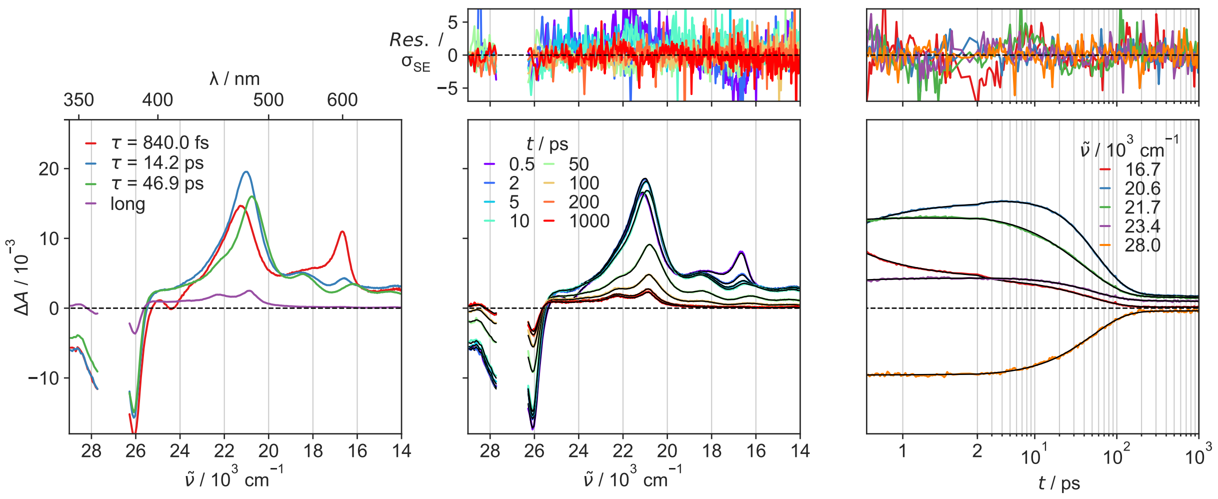

Figure S20: Global lifetime analysis of the UV-Vis transient absorption data measured with **IIIa** in hexafluoropropanol on the fs-ps timescale upon excitation at 375 nm. Left panel, evolution-associated difference spectra (EADS) and respective time constants. Middle and right panels, comparison of the best-fit (black line) and the spectra and time profiles at given time delays and wavenumbers, respectively.

S9.14 fs-ps Vis-TA of IIIs in ACN

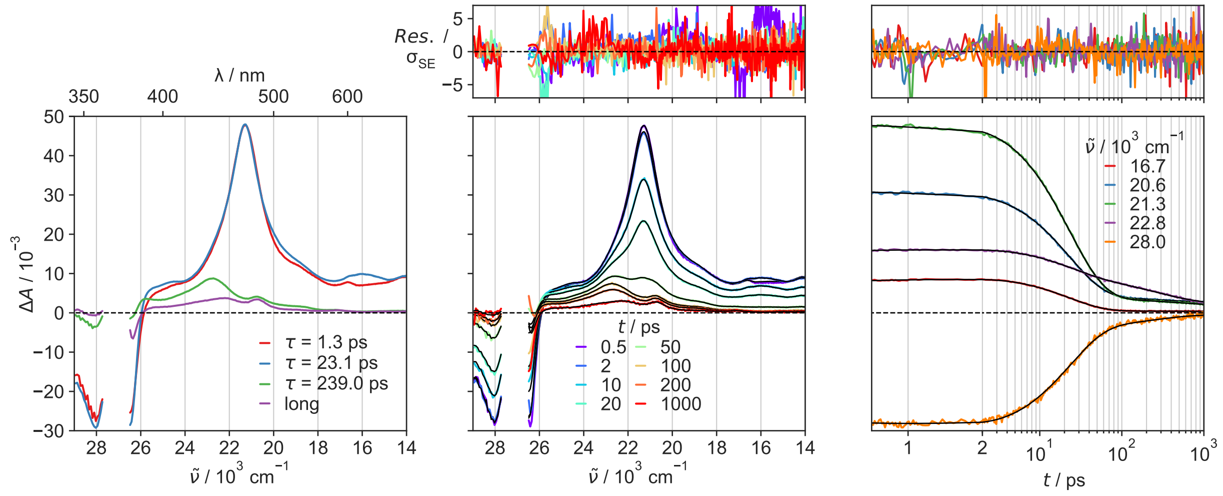

Figure S21: Global lifetime analysis of the mid-IR transient absorption data measured with **IIIs** in acetonitrile on the fs-ps timescale upon excitation at 375 nm. Left panel, evolution-associated difference spectra (EADS) and respective time constants. Middle and right panels, comparison of the best-fit (black line) and the spectra and time profiles at given time delays and wavenumbers, respectively.

S9.15 fs-ps Vis-TA of IVa in ACN

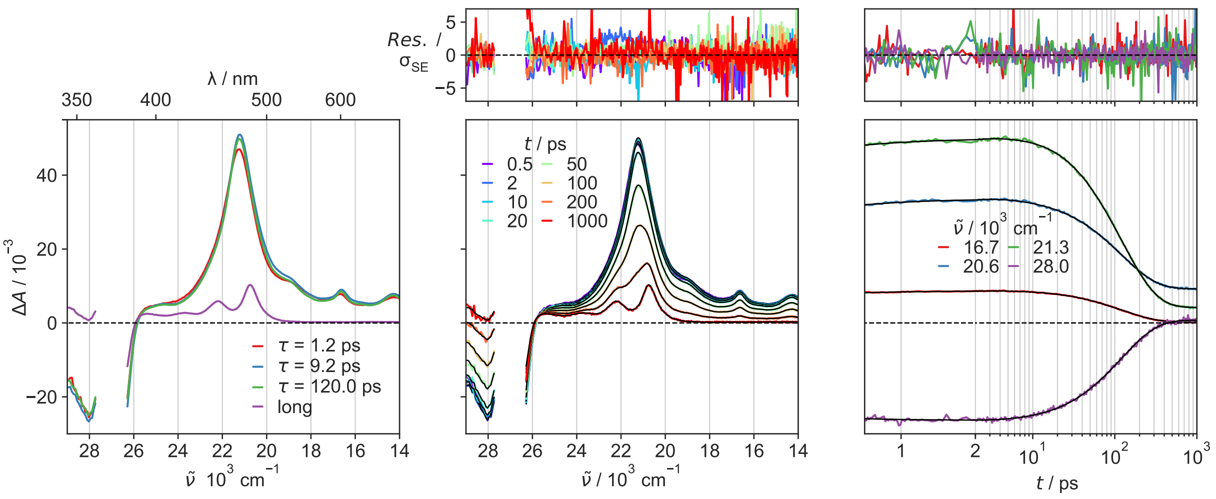

Figure S22: Global lifetime analysis of the mid-IR transient absorption data measured with **IVa** in acetonitrile on the fs-ps timescale upon excitation at 375 nm. Left panel, evolution-associated difference spectra (EADS) and respective time constants. Middle and right panels, comparison of the best-fit (black line) and the spectra and time profiles at given time delays and wavenumbers, respectively.

S9.16 fs-ps IR-TA of IVa in ACN

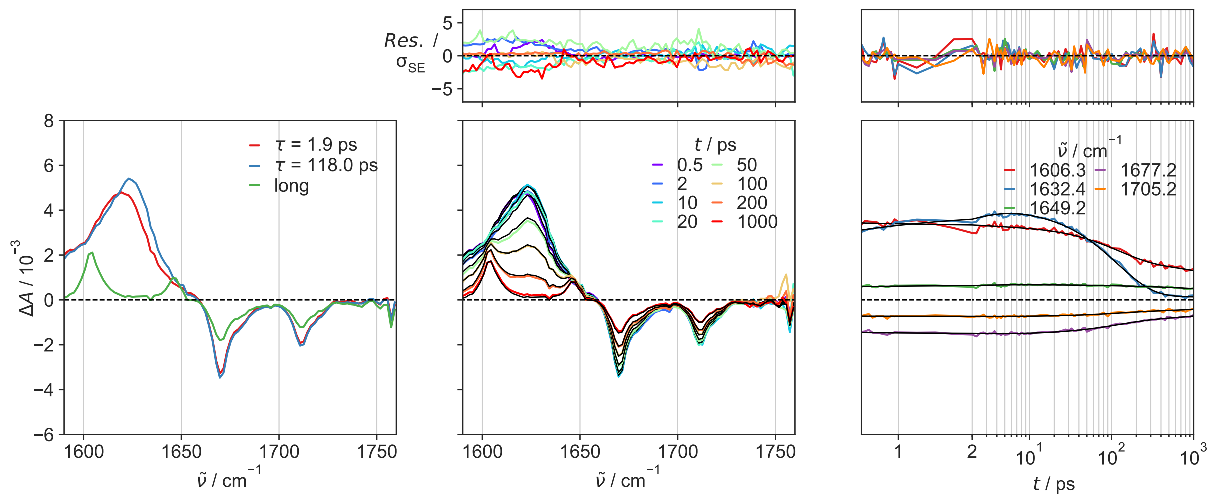

Figure S23: Global lifetime analysis of the mid-IR transient absorption data measured with **IVa** in acetonitrile on the fs-ps timescale upon excitation at 375 nm. Left panel, evolution-associated difference spectra (EADS) and respective time constants. Middle and right panels, comparison of the best-fit (black line) and the spectra and time profiles at given time delays and wavenumbers, respectively.

S9.17 fs-ps Vis-TA of IVa in MCH

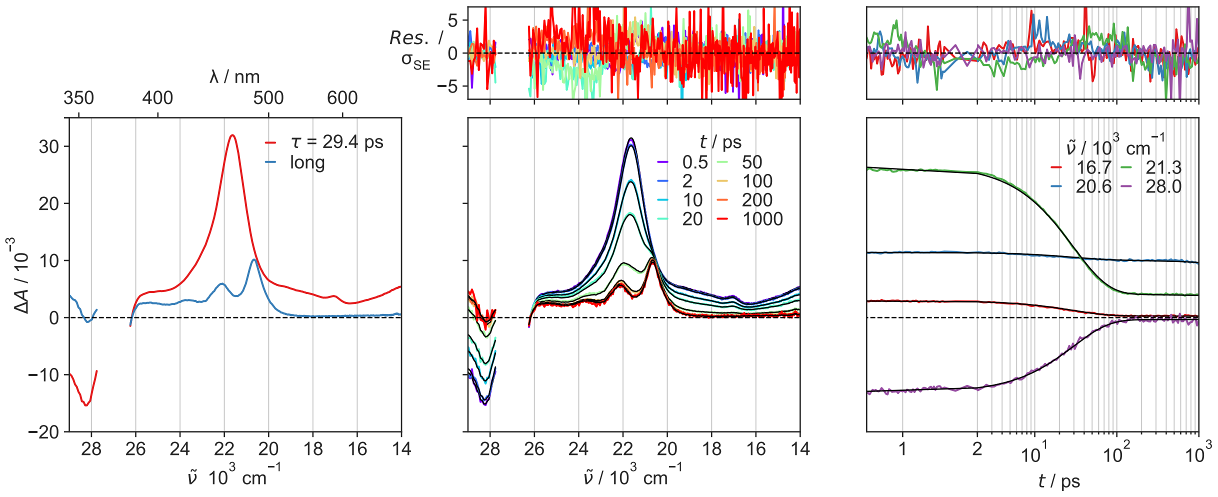

Figure S24: Global lifetime analysis of the mid-IR transient absorption data measured with **IVa** in methylcyclohexane on the fs-ps timescale upon excitation at 375 nm. Left panel, evolution-associated difference spectra (EADS) and respective time constants. Middle and right panels, comparison of the best-fit (black line) and the spectra and time profiles at given time delays and wavenumbers, respectively.

S9.18 fs-ps IR-TA of IVa in MCH

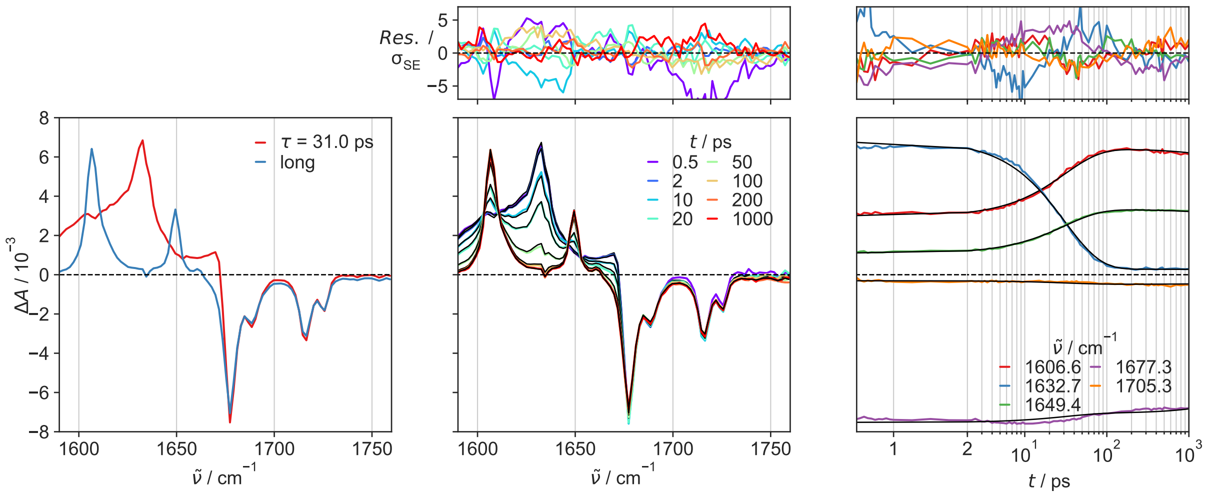

Figure S25: Global lifetime analysis of the mid-IR transient absorption data measured with **IVa** in methylcyclohexane on the fs-ps timescale upon excitation at 375 nm. Left panel, evolution-associated difference spectra (EADS) and respective time constants. Middle and right panels, comparison of the best-fit (black line) and the spectra and time profiles at given time delays and wavenumbers, respectively..

S9.19 fs-ps Vis-TA of IVs in ACN

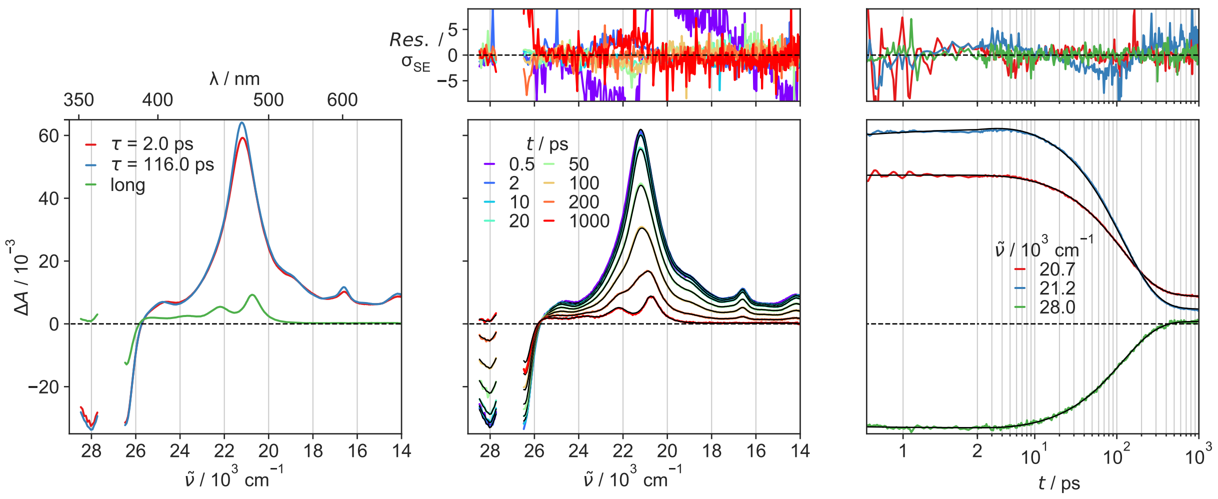

Figure S26: Global lifetime analysis of the mid-IR transient absorption data measured with **IVs** in acetonitrile on the fs-ps timescale upon excitation at 375 nm. Left panel, evolution-associated difference spectra (EADS) and respective time constants. Middle and right panels, comparison of the best-fit (black line) and the spectra and time profiles at given time delays and wavenumbers, respectively.

S9.20 fs-ps Vis-TA of IVs in MCH

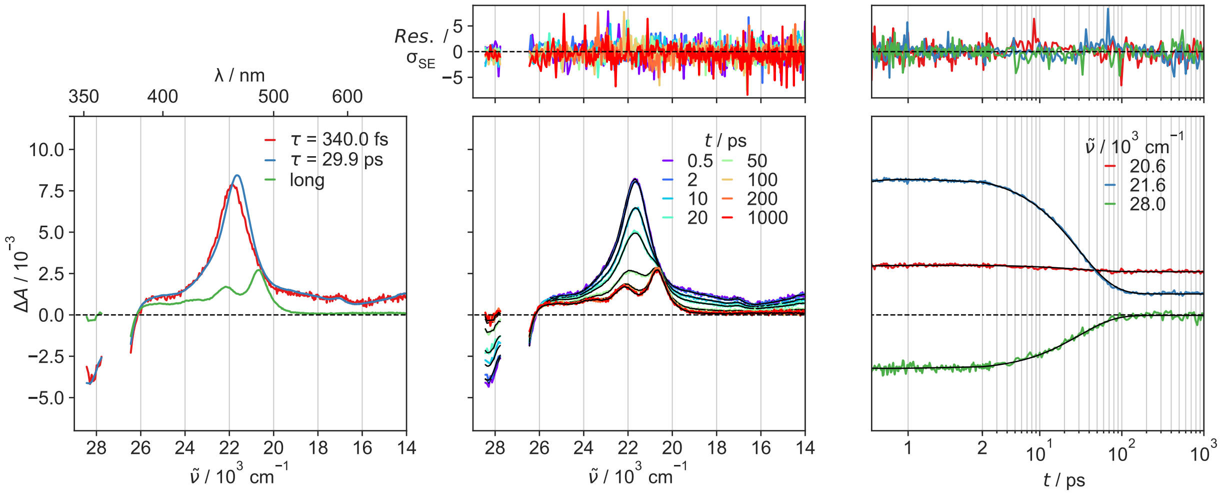

Figure S27: Global lifetime analysis of the mid-IR transient absorption data measured with **IVs** in methylcyclohexane on the fs-ps timescale upon excitation at 375 nm. Left panel, evolution-associated difference spectra (EADS) and respective time constants. Middle and right panels, comparison of the best-fit (black line) and the spectra and time profiles at given time delays and wavenumbers, respectively.

S9.21 fs-ps Vis-TA of Vs in ACN

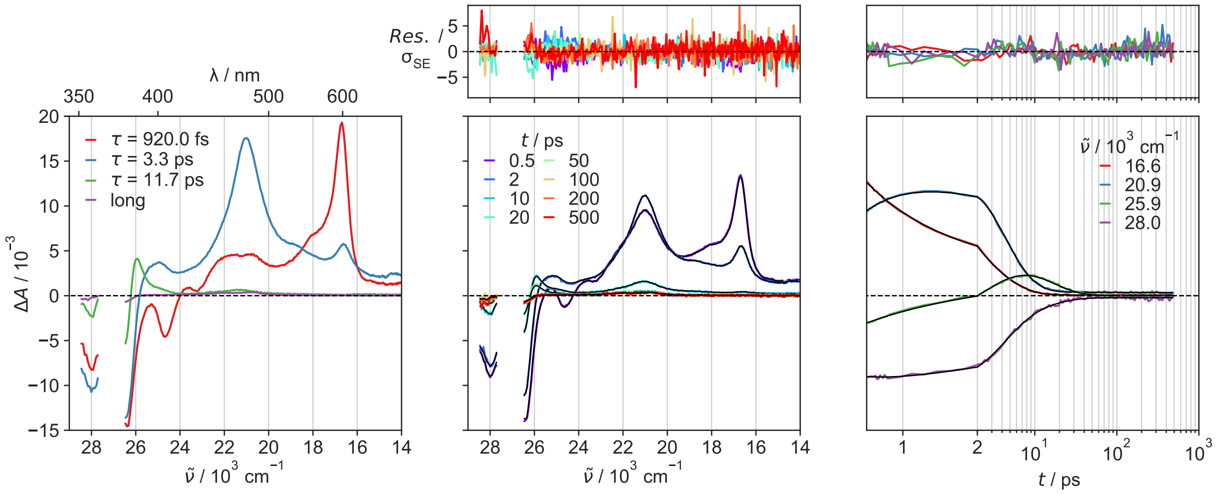

Figure S28: Global lifetime analysis of the mid-IR transient absorption data measured with **Vs** in acetonitrile on the fs-ps timescale upon excitation at 375 nm. Left panel, evolution-associated difference spectra (EADS) and respective time constants. Middle and right panels, comparison of the best-fit (black line) and the spectra and time profiles at given time delays and wavenumbers, respectively.

S9.22 fs-ps Vis-TA of Vs in THF

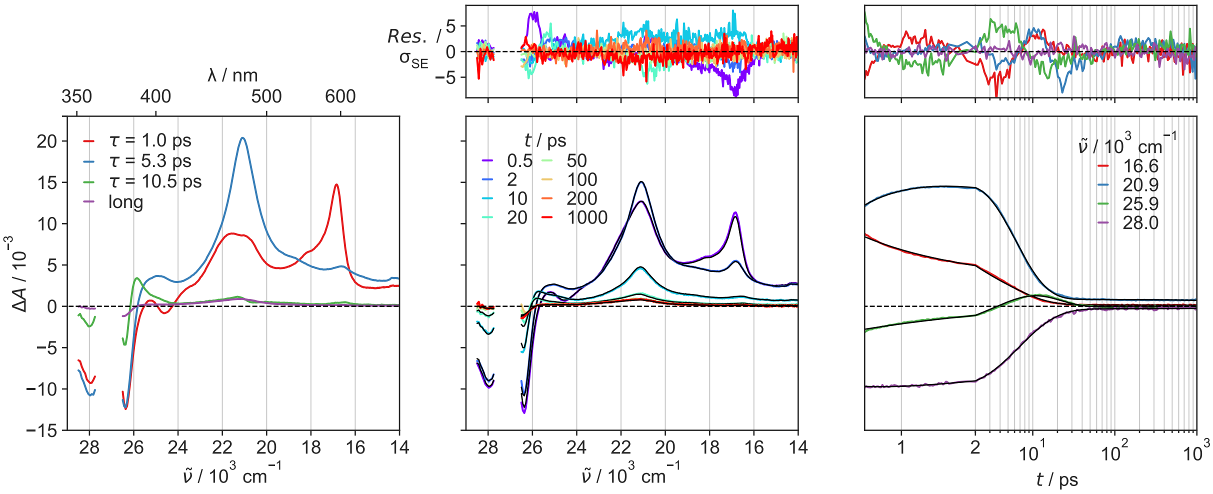

Figure S29: Global lifetime analysis of the mid-IR transient absorption data measured with **Vs** in THF on the fs-ps timescale upon excitation at 375 nm. Left panel, evolution-associated difference spectra (EADS) and respective time constants. Middle and right panels, comparison of the best-fit (black line) and the spectra and time profiles at given time delays and wavenumbers, respectively.

S9.23 fs-ps Vis-TA of Cage in THF

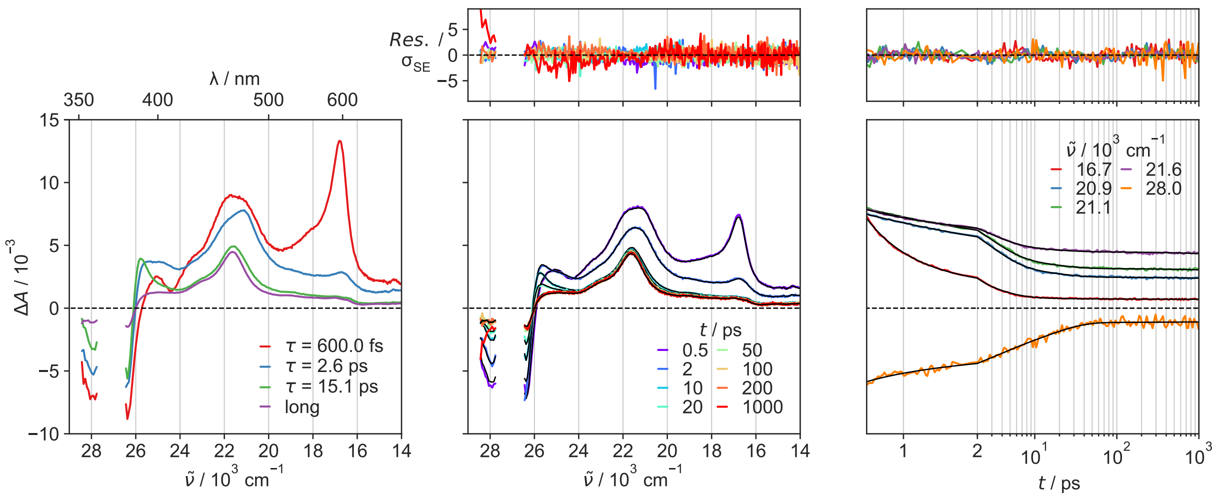

Figure S30: Global lifetime analysis of the mid-IR transient absorption data measured with **Cage** in THF on the fs-ps timescale upon excitation at 375 nm. Left panel, evolution-associated difference spectra (EADS) and respective time constants. Middle and right panels, comparison of the best-fit (black line) and the spectra and time profiles at given time delays and wavenumbers, respectively.

S10 ns-µs Vis-TA of Cage and Vs in THF

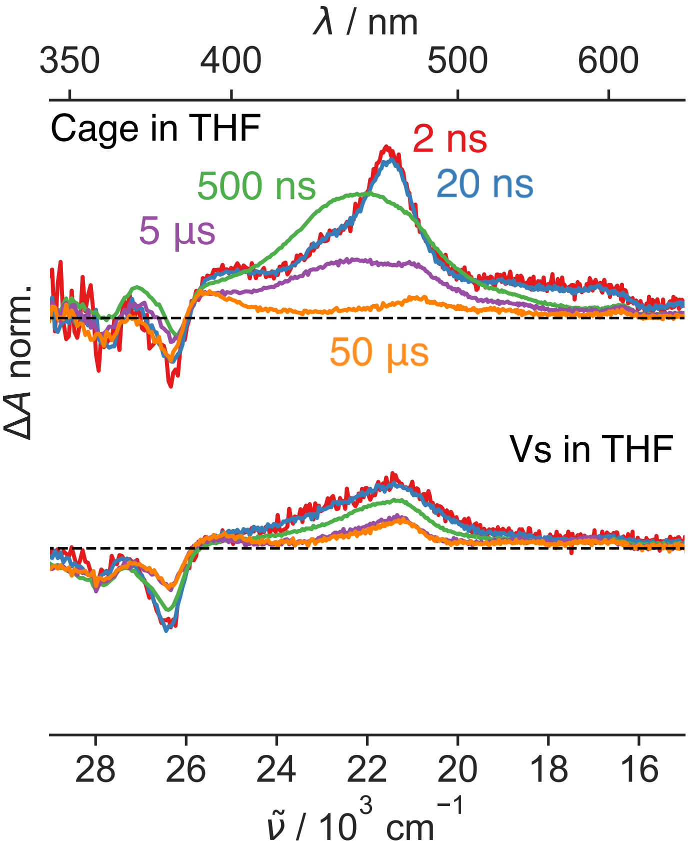

Figure S31: Transient absorption spectra of **Cage** (top) and **Vs** (bottom), at various time delays, upon excitation at 355 nm. The origin of the band appearing at 500 ns with **Cage** and the residual signal at 50 µs remain unknown. Since these features are not observed with **Vs** they are most likely linked to the electron-donor bridge and could originate from an intermolecular reaction. The decay of the ^{3}CSS of **Vs** is bimodal, with the slowest part appearing as a residual signal at 50 µs.

References

[1] P. Ganesan, X. Yang, J. Loos, T. J. Savenije, R. D. Abellon, H. Zuilhof and E. J. R. Sudhölter, *J. Am. Chem. Soc.*, 2005, **127**, 14530–14531.

[2] A. J. Taylor, E. S. Davies, J. A. Weinstein, I. V. Sazanovich, O. V. Bouganov, S. A. Tikhomirov, M. Towrie, J. McMaster and C. D. Garner, *Inorg. Chem.*, 2012, **51**, 13181–13194.

[3] R. E. Dawson, A. Hennig, D. P. Weimann, D. Emery, V. Ravikumar, J. Montenegro, T. Takeuchi, S. Gabutti, M. Mayor, J. Mareda, C. A. Schalley and S. Matile, *Nat. Chem.*, 2010, **2**, 533–538.

[4] A. Aster, A.-B. Bornhof, N. Sakai, S. Matile and E. Vauthey, submitted.

[5] A.-B. Bornhof, A. Bauzá, A. Aster, M. Pupier, A. Frontera, E. Vauthey, N. Sakai and S. Matile, *J. Am. Chem. Soc.*, 2018, **140**, 4884–4892.

[6] T. Šolomek, N. E. Powers-Riggs, Y.-L. Wu, R. M. Young, M. D. Krzyaniak, N. E. Horwitz and M. R. Wasielewski, *J. Am. Chem. Soc.*, 2017, **139**, 3348–3351.

[7] B. Lang, *Rev. Sci. Instrum.*, 2018, **89**, 093112.

[8] N. Banerji, G. Duvanel, A. Perez-Velasco, S. Maity, N. Sakai, S. Matile and E. Vauthey, *J. Phys. Chem. A*, 2009, **113**, 8202–8212.

[9] B. Lang, S. Mosquera-Vázquez, D. Lovy, P. Sherin, V. Markovic and E. Vauthey, *Rev. Sci. Instrum.*, 2013, **84**, 073107.

[10] E. Tokunaga, A. Terasaki and T. Kobayashi, *J. Opt. Soc. Am. B*, 1996, **13**, 496–513.

[11] M. Koch, R. Letrun and E. Vauthey, *J. Am. Chem. Soc.*, 2014, **136**, 4066–4074.

[12] J. S. Beckwith, C. A. Rumble and E. Vauthey, *Int. Rev. Phys. Chem.*, 2020, **39**, 135–216.
